# A whole‐genome scan for Artemisinin cytotoxicity reveals a novel therapy for human brain tumors

**DOI:** 10.15252/emmm.202216959

**Published:** 2023-02-06

**Authors:** Jasmin Taubenschmid‐Stowers, Michael Orthofer, Anna Laemmerer, Christian Krauditsch, Marianna Rózsová, Christian Studer, Daniela Lötsch, Johannes Gojo, Lisa Gabler, Matheus Dyczynski, Thomas Efferth, Astrid Hagelkruys, Georg Widhalm, Andreas Peyrl, Sabine Spiegl‐Kreinecker, Dominic Hoepfner, Shan Bian, Walter Berger, Juergen A Knoblich, Ulrich Elling, Moritz Horn, Josef M Penninger

**Affiliations:** ^1^ IMBA, Institute of Molecular Biotechnology of the Austrian Academy of Sciences Vienna Biocenter Vienna Austria; ^2^ JLP Health GmbH Vienna Austria; ^3^ Center for Cancer Research and Comprehensive Cancer Center‐Central Nervous System Tumor Unit Medical University of Vienna Vienna Austria; ^4^ Department of Pediatrics and Adolescent Medicine and Comprehensive Center for Pediatrics Medical University of Vienna Vienna Austria; ^5^ Novartis Institutes for BioMedical Research Basel Switzerland; ^6^ Department of Neurosurgery Medical University Vienna Vienna Austria; ^7^ Department of Pediatric Oncology Dana‐Farber Boston Children's Cancer and Blood Disorders Center Boston MA USA; ^8^ Broad Institute of Harvard and MIT Cambridge MA USA; ^9^ Department of Pharmaceutical Biology, Institute of Pharmaceutical and Biomedical Sciences Johannes Gutenberg University Mainz Germany; ^10^ Department of Neurosurgery, Kepler University Hospital GmbH Johannes Kepler University Linz Linz Austria; ^11^ Institute for Regenerative Medicine, Shanghai East Hospital, School of Life Sciences and Technology Tongji University Shanghai China; ^12^ Frontier Science Center for Stem Cell Research Tongji University Shanghai China; ^13^ Department of Medical Genetics, Life Sciences Institute University of British Columbia Vancouver BC Canada; ^14^ Present address: Epigenetics Programme Babraham Institute Cambridge UK; ^15^ Present address: Altos Labs Cambridge Institute of Science Cambridge UK

**Keywords:** 5‐ALA, Artemisinin, genome wide screen, glioblastoma therapy, porphyrin biogenesis, Cancer, Pharmacology & Drug Discovery

## Abstract

The natural compound Artemisinin is the most widely used antimalarial drug worldwide. Based on its cytotoxicity, it is also used for anticancer therapy. Artemisinin and its derivates are endoperoxides that damage proteins in eukaryotic cells; their definite mechanism of action and host cell targets, however, have remained largely elusive. Using yeast and haploid stem cell screening, we demonstrate that a single cellular pathway, namely porphyrin (heme) biosynthesis, is required for the cytotoxicity of Artemisinins. Genetic or pharmacological modulation of porphyrin production is sufficient to alter its cytotoxicity in eukaryotic cells. Using multiple model systems of human brain tumor development, such as cerebral glioblastoma organoids, and patient‐derived tumor spheroids, we sensitize cancer cells to dihydroartemisinin using the clinically approved porphyrin enhancer and surgical fluorescence marker 5‐aminolevulinic acid, 5‐ALA. A combination treatment of Artemisinins and 5‐ALA markedly and specifically killed brain tumor cells in all model systems tested, including orthotopic patient‐derived xenografts *in vivo*. These data uncover the critical molecular pathway for Artemisinin cytotoxicity and a sensitization strategy to treat different brain tumors, including drug‐resistant human glioblastomas.

## Graphical abstract

Artemisinin requires cellular heme for its cytotoxic function. Its cytotoxicity can be increased by the porphyrin enhancer 5‐ALA, which accumulates in cancer cells. 5‐ALA sensitizes tumor cell.

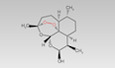

## Introduction

*Artemisia annua*, or *sweet wormwood*, extracts have been used in traditional Chinese medicine for thousands of years to treat fever, colds, and other maladies (Efferth, 2017). In the 1970s, the active compound of *Artemisia annua*, called Artemisinin, was first isolated and described as an antimalarial drug by the Chinese scientist Tu Youyou, who was awarded the Nobel Prize in Medicine in 2015 for its discovery. Artemisinin, and its derivatives such as dihydroartemisinin (DHA) or artesunate (ARS), is one of the most commonly used antimalarial drugs worldwide (Klayman, 1985; Bosman & Mendis, 2007). It kills the unicellular parasite *P. falciparum* that causes malaria, in all of its life stages (Aweeka & German, 2008). More recently, potent anticancer properties of Artemisinin have been recognized (Krishna *et al*, 2008). The variety of the different functions of Artemisinin and its applications have pointed toward a wide‐ranging mechanism of action in eukaryotic cells.

Biochemically, Artemisinin is an endoperoxide. Its endoperoxide properties are required for its antimalarial effect (Meshnick *et al*, 1996). Once the endoperoxide bridge is being cleaved, Artemisinin gets activated and leads to the production of reactive oxygen species (ROS) and free radicals, which subsequently alkylate susceptible proteins and macromolecules in the cell. This alkylation reaction alters the structure and function of proteins, damages DNA, and induces cellular stress, eventually resulting in cell death. In addition, it has been reported that Artemisinin associates with a wide range of cellular proteins thereby affecting multiple pathways, including glycolysis, protein biosynthesis, mitochondrial processes, and antioxidant responses (Zhang *et al*, 2015; Ismail *et al*, 2016). While definite regulators or activators of Artemisinin are still being debated, free or complexed iron, such as in haem of hemoglobin, are considered strong candidates. However, Artemisinin's promiscuous binding properties, together with the high number of reported functions, have so far hindered the identification of its definite cellular targets (Mbengue *et al*, 2015; Straimer *et al*, 2015; Tilley *et al*, 2016). As an increasing number of Artemisinin‐resistant malaria cases are surfacing and at high doses, Artemisinin exerts strong cytotoxic activity, it is imperative to delineate the compound's mechanism of action.

To identify the essential endogenous Artemisinin targets in eukaryotic cells, we employed two independent, genetically tractable screening systems: HIP‐HOP profiling in *S*. *cerevisiae* and haploid mutagenesis in mouse embryonic stem cells (ESCs). Using genome‐wide screens, we identified mitochondrial function and specifically, porphyrin biosynthesis, as the essential pathway for dihydroartemisinin toxicity in eukaryotic cells. Genetic and pharmacological manipulation of porphyrin production was sufficient to alter the sensitivity of multiple murine and human tumor cells to DHA *in vitro*. Sensitization of this pathway using the clinically approved photodynamic porphyrin enhancer and surgical fluorescence marker 5‐ALA markedly increased the anticancer activity of Artemisinin and its derivatives not just in human cerebral tumor organoids and patient‐derived brain tumor spheroids but also in orthotopic patient‐derived xenografts *in vivo*—thus identifying a novel treatment option for yet largely untreatable brain tumors such as glioblastomas.

## Results

### HIP‐HOP yeast screens identify porphyrin biosynthesis as target for Artemisinin toxicity

**Figure 1:**
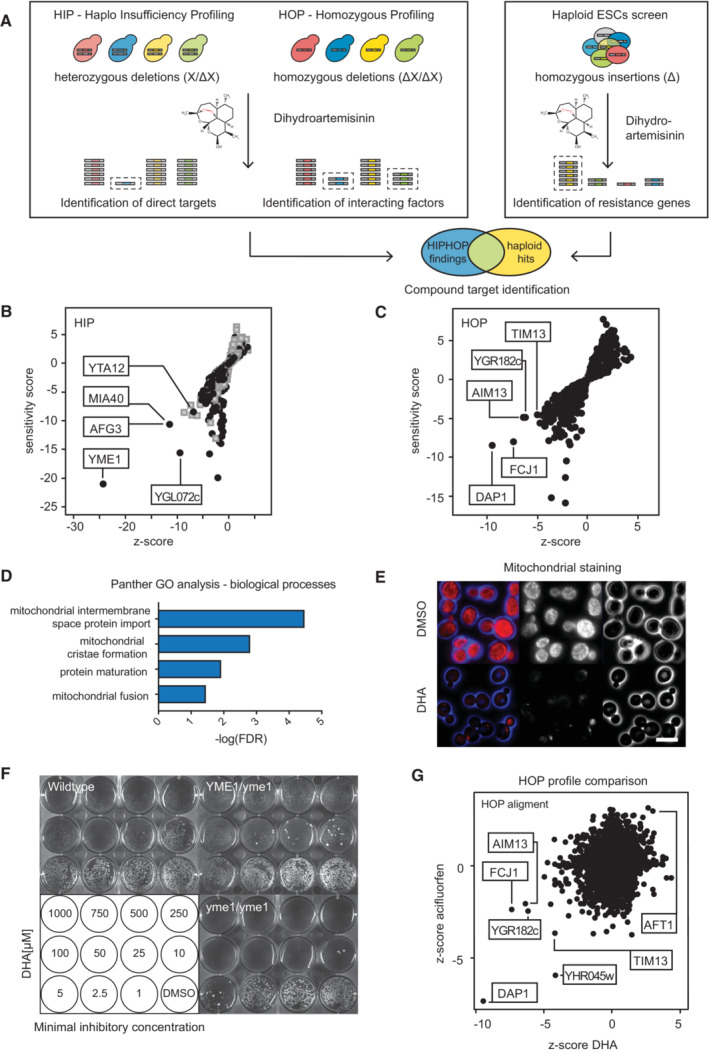
Mitochondria are key modulators of dihydroartemisinin toxicity in yeast A
Schematic of yeast HIP‐HOP and haploid embryonic stem cell screening for compound target identification.B
Haploinsufficiency profiling (HIP) of a yeast deletion collection at sublethal concentrations (IC_30_, 3 μM) of dihydroartemisinin (DHA). Profile is plotted by sensitivity and z‐score (i.e., relevance, for more information see materials and methods). Strains with essential genes are indicated in gray, relevant mutants of nonessential genes (black) are labeled.C
Homozygous profile (HOP) of the yeast deletion collection with DHA (IC_10,_ 10 μM). Sensitivity and z‐scores are plotted.D
GO‐term analysis of HIP‐HOP hits.E
Mitochondrial (MitoTracker) staining of DMSO (upper panel) or DHA (lower panel) treated cells. Scale bar 5 μm.F
Minimal inhibitory concentrations of DHA treated wild‐type, heterozygous, (*YME1*/*yme1*) and homozygous (*yme1*/*yme1*) mutant strains. Plates were imaged after 72 h.G
HOP profile alignment of z‐scores (significances) of DHA and acifluorfen. Z‐scores couple the sensitivity score of a strain in a given profile to the variability in sensitivity of that strain across all the compounds tested.

To identify factors and pathways required for Artemisinin activity in eukaryotic cells in an unbiased manner, we used two independent, genetically tractable model systems for high‐throughput screening: We first employed HIP‐HOP chemogenomic phenotypic profiling in yeast (*Saccharomyces cerevisiae*), and second, insertional mutagenesis and forward genetic screening in haploid mouse ESCs (Fig 1A;Elling *et al*, 2011; Hoepfner *et al*, 2014). Chemogenomic approaches in yeast have identified primary targets or mechanism of action of compounds, for instance, finding mTOR as a Rapamycin target (Roemer *et al*, 2011a). Haploinsufficiency profiling (HIP) exploits the increased sensitivity toward a compound after lowering the dosage of the target‐encoding gene from two copies to one copy in diploid yeast. In the complementary approach, homozygous deletion profiling (HOP), both copies of nonessential genes are deleted, identifying synthetic lethality upon target inhibition (Giaever *et al*, 1999; Roemer *et al*, 2011b; Hoepfner *et al*, 2014).

**Figure EV1:**
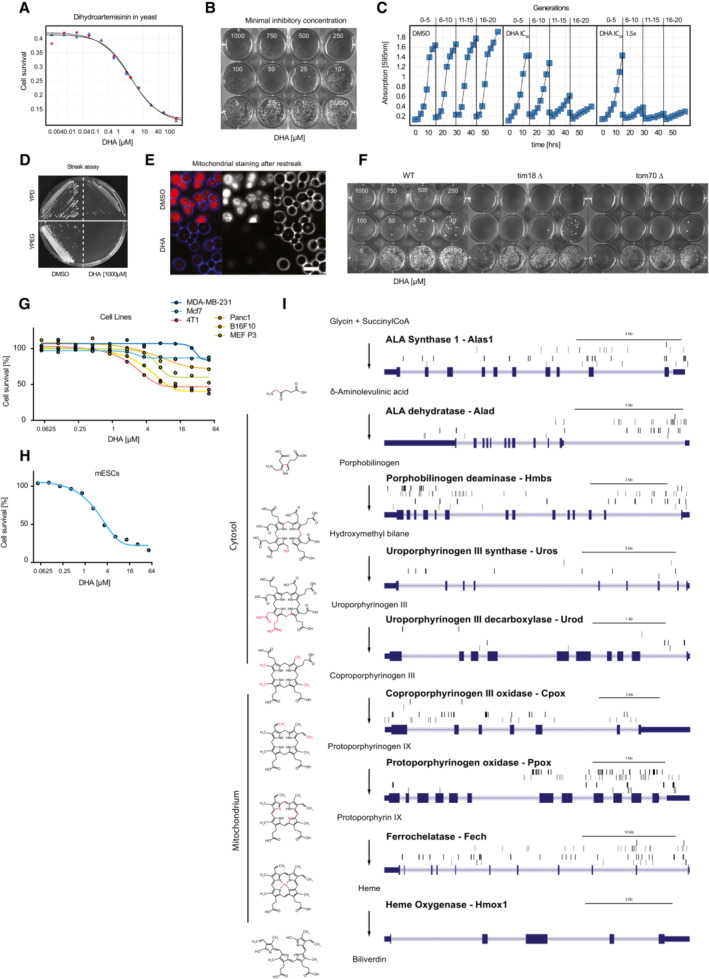
Identification of essential host cell factors for dihydroartemisinin cytotoxicity in yeast and murine ESCs A
IC_50_ curves of yeast cells grown in two different batches of Artemisinin. Batch one was freshly added from the DMSO stock solution before the start of the experiment (triangles), the other one was added 60 h before and stored at room temperature (stars). The survival curves and IC_50_ values of treated cells are indistinguishable.B
Minimal inhibitory concentration (MIC) of dihydroartemisinin (DHA) on solid medium. Approximately 1,000 haploid wild‐type strain cells were plated per well at different concentrations of DHA and imaged after 72 h.C
HIP pool growth curves over 20 cell generations with three back dilutions. Whereas DMSO control cells divide at similar rates, cells with 1× or 1.5× IC_30_ concentrations of DHA show reduced proliferation after each back dilution. Absorption was measured at 595 nm.D
Colony formation assay of DHA‐treated yeast cells on YPD and YPEG plates. Cells were pretreated with DMSO or 1,000 μM DHA on solid medium and re‐streaked onto glucose (YPD, fermentable carbon source) or ethanol/glycerol containing medium (YPEG, nonfermentable carbon source).E
Mitochondrial (MitoTracker) staining of DMSO (upper panel) or DHA (1,000 μM, lower panel) pretreated cells (after 72 h recovery on YPD). Scale bar 5 μm.F
MIC determination of DHA in homozygous and heterozygous tim18 and tom70 mutant yeast clones. Approximately 1,000 cells were plated per well, treated for 72 h and imaged.G, H
Cell survival of DHA‐treated (G) mouse ESCs and (H) primary mouse fibroblasts (MEF p3), as well as mouse (B16F10—skin melanoma, 4T1—breast cancer) and human (MDA‐MB‐231—triple negative epithelial breast cancer, Mcf7—estrogen‐/progesterone receptor‐positive/HER2‐negative breast cancer, Panc1—pancreatic) cancer cells. Viability was assessed after 48 h of treatment using Alamar Blue staining.I
Porphyrin biosynthesis pathway component integrations sites from the DHA screens in haploid ESCs. Genomic locations and targeted introns, exons of porphyrin biosynthesis genes (i.e., enzymes, bold), as well as retroviral (upper panel) or Tol2 transposon (lower panel) integrations sites (vertical bars) are shown on the forward (black) and reverse (gray) strands. The chemical structures of the substrates and products are shown for each enzyme reaction.

Haploinsufficiency profiling was performed as previously described (Hoepfner *et al*, 2014). The sensitivity of diploid wild‐type yeast strains to dihydroartemisinin was assessed based on growth rates in the presence of the compound (Fig EV1A–C). HIP profiling of dihydroartemisinin in yeast revealed a single, prominent hit—the mitochondrial inner membrane protease complex (iAAA) subunit Yme1 (Fig 1B). Yme1 is required for mitochondrial protein folding and degradation, as well as its structure, turnover, and function (Dunn *et al*, 2006). Additional genes identified from the dihydroartemisinin HIP profile included the second major protease complex (mAAA) constituents Yta10 (AFG3) and Yta12 (RCA1) (Arlt *et al*, 1996) and the mitochondrial intermembrane space import and assembly protein 40, Mia40 (Morales *et al*, 1992; Chacinska *et al*, 2004) (Fig 1B).

Homozygous profiling (HOP) of yeast mutants was performed under similar conditions as HIP but based on growth rates of haploid strains. Sensitivity and relevance analysis identified Dap1 as the strongest growth altering factor in the presence of dihydroartemisinin (Fig 1C). Dap1 is a heme‐binding protein that is involved in the regulation of cytochrome P450 and DNA damage responses (Craven *et al*, 2007). The HOP profile also included several mitochondrial factors such as the mitochondrial inner membrane complex proteins Fcj1 (MIC60) and Aim13 (MIC19), the mitochondrial membrane chaperone Tim13 and the overlapping ORF *YGR182c*. Analysis for abundant cellular processes in both the HIP and HOP yeast profiles by GO‐term analysis confirmed strong enrichment for genes involved in mitochondrial processes (Fig 1D).

Notably, profiling of wild‐type yeast strains revealed marked colony growth differences in the presence of Artemisinin. High concentrations of the compound induced the appearance of small yeast colonies that displayed increased resistance to dihydroartemisinin (Fig EV1B). Dihydroartemisinin treated wild‐type yeast showed normal budding and growth phenotypes on fermentable (YPD, glucose) carbon sources (Fig EV1C), but were not viable on nonfermentable (YPEG, ethanol/glycerol) carbon sources (Fig EV1D). Additionally, such small colonies had lost their functional mitochondria (Nunnari & Walter, 1996; Fig 1E; Fig EV1E). These growth properties and deficient phenotypes are reminiscent of yeast petite mutants that display smaller colony size and oxidative phosphorylation defects, preventing them to grow on nonfermentable carbon sources.

Our most significant HIP hit, YME1, has been previously described as petite‐negative (thus unable to form such small colonies) and highly dependent on intact mitochondria even in the presence of fermentable carbon sources (Thorsness *et al*, 1993). The identification of the YME1 strain in the HIP profile together with the phenotype of reduced growth rates in the presence of dihydroartemisinin (Fig 1F) suggested a general hypersensitivity of petite‐minus strains to the compound. We thus assessed the growth rates of two other well‐characterized yeast strains that are deficient for the mitochondrial import proteins Tom70 and Tim18, upon dihydroartemisinin treatment. Both mutant strains failed to form petites (i.e., small yeast colonies) resulting in higher susceptibility to the antimalarial compound (Dunn *et al*, 2006; Fig EV1F), thus confirming the requirement of functional mitochondria for dihydroartemisinin toxicity in yeast. Lastly, comparison of the dihydroartemisinin HOP profile to ~ 4,000 independent compound analyses in the HIP‐HOP chemogenomics database identified acifluorfen (Matringe *et al*, 1989) as the most analogous substance to the antimalarial compound (Fig 1G). Acifluorfen is an inhibitor of protoporphyrinogen oxidase, PPOX, an inner mitochondrial membrane enzyme that is involved in porphyrin or heme production in cells and targets the same set of mitochondrial proteins as those that were found by our dihydroartemisinin screen (Tim13, Aim13 (MIC19), Fcj1 (MIC60), and Ubx2) (Witkowski & Halling, 1989). These results point toward a specific role of mitochondrial porphyrin production in Artemisinin toxicity in yeast.

### Haploid stem cell screens identify porphyrin biosynthesis as target for Artemisinin toxicity

**Figure 2:**
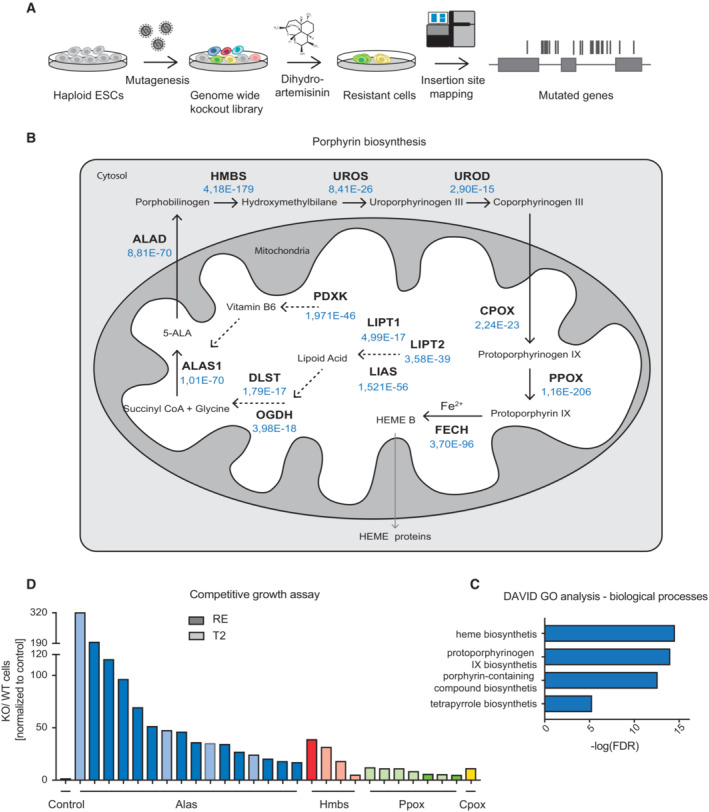
Haploid screening in mouse stem cells delineates porphyrin biosynthesis as an essential prerequisite for dihydroartemisinin toxicity A
Schematic of haploid embryonic stem cell screens for compound target identification.B
Porphyrin biogenesis pathway. Subcellular location (cytosol, mitochondria) and loss‐of‐function scores (italic blue) of major enzymes (bold, capitalized) and co‐factors (normal, capitalized) of retroviral mutagenesis screen are indicated. 5‐aminolevulinic acid (5‐ALA), ALA synthase 1 (ALAS1), ALA dehydratase (ALAD), porphobilinogen deaminase (HMBS), uroporphyrinogen III synthase (UROS), uroporphyrinogen III decarboxylase (UROD), coproporphyrinogen III oxidase (CPOX), protoporphyrinogen oxidase (PPOX), ferrochelatase (FECH), pyridoxal kinase (PDXK), lipoyltransferase 1, 2 (LIPT1, LITP2), lipoic acid synthetase (LIAS), oxoglutarate dehydrogenase (OGDH), dihydrolipoamide S‐succinyltransferase (DLST).C
GO‐term analysis reveals porphyrin biogenesis as the major pathway targeted by DHA.D
Competitive growth assay of DHA‐resistant single cell clones. Wild‐type (mCherry^+^_Cre_Puro) and knockout (GFP^+^_Puro) sister clones were derived from mutant (retrovirus—intron RE, darker shading, Tol2 transposon—intron T2, lighter shading) resistant colonies, treated with DHA, analyzed with flow cytometry and Sanger sequenced for insertion site mapping.

We conducted a reciprocal screen to identify targets of dihydroartemisinin in haploid murine stem cells (haESC) using genome‐wide mutagenesis (Fig 2A). In order to establish screening conditions, we determined LD values (lethal dosage) for the compound in wild‐type haploid ESCs and assessed growth rates in different murine and human tumor cell lines (Fig EV1G and H). For the generation of genome‐wide mutant ESC libraries, we used two targeting systems for insertional mutagenesis: Retrovirus and Tol2 transposons (Schnütgen *et al*, 2008; Elling *et al*, 2011). Selection of those two independent libraries led to the identification and recovery of dihydroartemisinin‐resistant colonies from the mutagenized cell pools, but not from control cells. Upon expansion and mapping of their insertion sites, enrichment scores based on loss‐of‐function (LOF) analysis were determined (Datasets EV1 and EV2).

Haploid mutagenesis and dihydroartemisinin compound screening identified the enzyme, protoporphyrinogen oxidase, PPOX, as the top hit in both mutant libraries. PPOX is an inner mitochondrial membrane enzyme involved in cellular porphyrin or heme production. Its inhibitor, acifluorfen, also had the most similar HOP chemogenomic profile to dihydroartemisinin in yeast (Fig 1G). Intriguingly, genetic mutants of every single enzyme of the porphyrin biosynthesis pathway, both in mitochondria (Alas1, Cpox, Ppox, Fech) and the cytoplasm (Alad, Hmbs, Uros, Urod), were recovered from the screen, as well as essentially all relevant co‐factor‐producing enzymes (Lias, Ogdh, Dlst, Lipt1, Lipt2, Pdxk) and pathways feeding into the protoporphyrin or heme biosynthesis (Fig 2B; Fig EV1I; Datasets EV1 and EV2). GO term analysis of the dihydroartemisinin screening profile confirmed porphyrin biosynthesis as the only significantly targeted pathway in the context of dihydroartemisinin toxicity (Fig 2C). Thus, similar to HIP‐HOP chemogenomic profiling, forward genetic screening in haESCs delineates porphyrin biosynthesis as the definitive and essential pathway for dihydroartemisinin cytotoxicity.

To confirm the hits from the high‐throughput dihydroartemisinin screen, we tested single mutant ESC clones for their susceptibility to dihydroartemisinin. Resistant colonies were recovered from both mutant libraries from the screen and tested individually. We separately transduced cell lines with a Cre expression construct to invert the splice acceptor knockout cassette by recombination (Cre_Cherry_puroR) or maintained it in the original orientation (GFP‐puro). Sister cell lines were mixed, cultured together and their relative growth rates were monitored in the presence and absence of dihydroartemisinin in a competition assay. While the relative amounts of labeled cells in a mixed population of mutant and wildtype cells did not change over time in the absence of the compound, dihydroartemisinin exposure resulted in selective loss of wild‐type haploid ESCs (mCherry_Cre) and strong expansion of knockout sister cells (GFP) and thus a shift in the ratios of fluorescent cells. Insertion site mapping of resistant, competition assay validated cell lines by inverse PCR and Sanger sequencing confirmed disruption of porphyrin biosynthesis pathway components in those cells (Fig 2D). These results show that mutations of porphyrin biosynthesis pathway enzymes cause resistance to the cytotoxic effects of dihydroartemisinin.

### Porphyrin biosynthesis is sufficient to alter cellular sensitivity to Artemisinin toxicity

**Figure 3:**
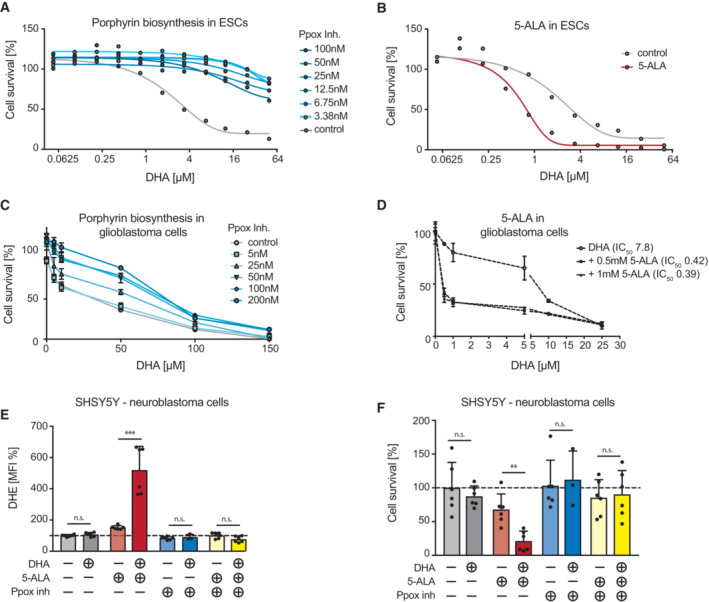
Modulation of porphyrin biosynthesis is sufficient to alter dihydroartemisinin toxicity via ROS generation A, B
Cell survival of dihydroartemisinin‐treated ESCs in combination with (A) the Ppox inhibitor acifluorfen or upon and (B) 5‐ALA administration. Alamar Blue staining was used to assess viability after 72 h of treatment.C, D
Cell survival of DHA treated primary human glioblastoma cells from cancer patients and in combination with (C) the Ppox inhibitor acifluorfen (BTL90 cells) or upon (D) 5‐ALA administration (VBT12 cells). Viability was assessed using CellTiter‐Glo after 72 h. Experiments were performed in triplicate. Values are mean ± SD.E, F
(E) ROS levels (DHE staining, PE 582/15 nm—MFI) and (F) cell survival of DHA (0.5 μM), 5‐ALA (0.25 mM) or Ppox inhibitor (10 μM) treated human neuroblastoma cells (SHSY5Y). Fluorescence of DHE (ROS levels) and cell numbers (survival) were assessed by flow cytometry and automated cell counting. The experiments were performed two times, in triplicate each. Values indicate mean ± SD. n.s. nonsignificant, **P < 0.01, ***P < 0.001, unpaired Student's *t*‐test.

**Figure EV2:**
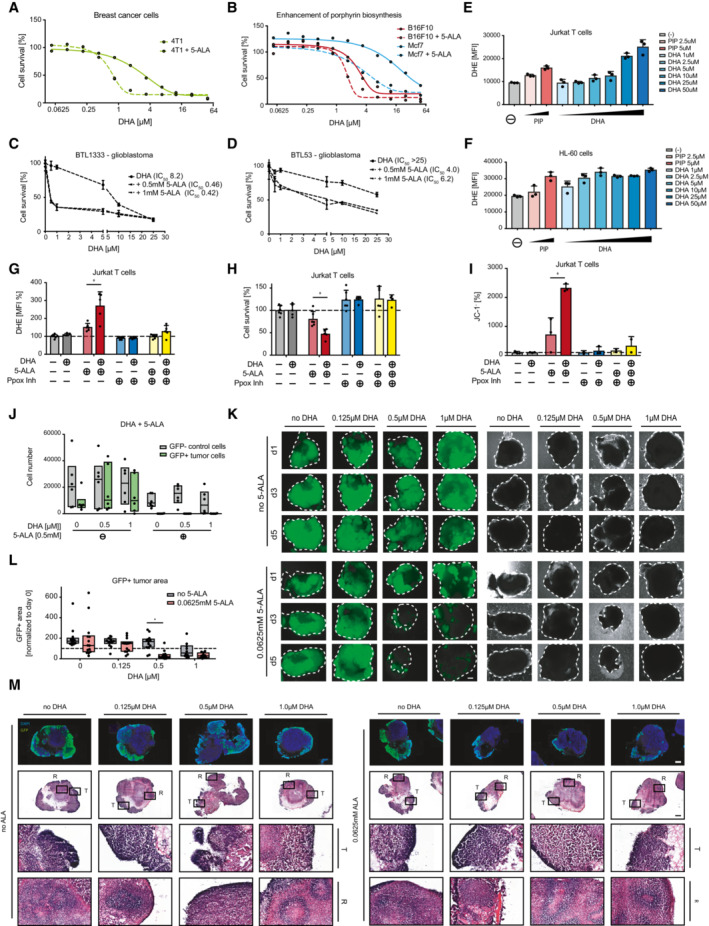
Modulation of porphyrin biosynthesis changes dihydroartemisinin cytotoxicity in cells and organoids A
Cell viability of DHA‐treated mouse cancer cells (4T1) in the presence and absence of 5‐ALA (0.5 mM). Alamar Blue was used to determine cell survival after 72 h of treatment.B
Cell survival of DHA treated mouse (B16F10) and human (Mcf7) cancer cells, in the presence and absence of 5‐ALA (0.5 mM). Alamar Blue was used to determine viability after 48 h.C, D
Cell survival of DHA treated primary human glioblastoma cells (BTL53, BTL1333) in the presence or absence of 5‐ALA. CellTiter‐Glo was used to assess viability, at 72 h. Experiment was performed in triplicate and repeated once. Values are mean ± SD.E, F
ROS/DHE staining and flow cytometry analysis of piperlongumine (PIP) or DHA (DHA) treated (E) Jurkat T cells and (F) HL‐60 cells (48 h). Experiments were performed in triplicate. Values are mean ± SD.G, H, I
(G) ROS levels (DHE staining, PE 582/15 nm—MFI), (H) cell survival and (I) JC‐1 levels in DHA (0.5 μM), 5‐ALA (0.25 mM), or Ppox inhibitor (10 μM) treated Jurkat T‐cells, 48 h. DHE fluorescence, relative cell numbers, and percentages of JC‐1‐negative cells were assessed using flow cytometry and automated high throughput cell counting. (G–I) All experiments were performed in triplicate and repeated once (JC‐1) or twice (DHE, cell survival). Values are means ± SD.J
Cell survival of dissociated tumor organoids (central nervous system primitive neuroectodermal tumor (CNS‐PNET‐like) neoplasm model, c‐MYC overexpression). Quantification of GFP^+^ tumor cells and GFP‐control cells of treated organoids normalized to control (DMSO) using flow cytometry are shown. Data are shown as box plots (25^th^–75^th^ percentiles, median). The experiment was performed in triplicate and repeated twice.K
Representative fluorescence (left panel) and brightfield (right panel) images of control (DMSO), 5‐ALA (0.0625 mM), DHA (1 μM), or 5‐ALA + DHA (0.0625 mM and 1 μM) treated cerebral tumor organoids. Scale bar 500 μm.L
Quantification of GFP‐positive tumor areas on day 5 of treatment, as compared to day 1. Box plots (25^th^–75^th^ percentiles, median) of data are shown. The experiments were performed in triplicate each, repeated five times. Student's *t*‐test was used to determine significance; *P < 0.05.M
Representative fluorescent (anti‐GFP, DAPI) and H&E (hematoxylin and eosin stained) images of fixed cryo‐sections of control and treated tumor organoids (CNS‐PNET‐like neoplasm). Regions of rosette‐like structures (R) and tumor tissue (T) are indicated and magnified (6.8×). Top panels show GFP‐labeled tumor cells (green) and DAPI counterstaining (blue) to image nuclei. Scale bar 500 μm.

To corroborate that modulation of cellular porphyrin biosynthesis is sufficient to alter susceptibility to dihydroartemisinin, we used a pharmacological inhibitor of porphyrin/heme biosynthesis, the protoporphyrinogen oxidase (PPOX) inhibitor acifluorfen, which blocks conversion of protoporphyrinogen IX to protoporphyrin IX. Treatment of mouse ESCs with acifluorfen markedly increased their resistance to dihydroartemisinin (Fig 3A; Witkowski & Halling, 1989). Conversely, when we enhanced porphyrin production using the nonproteinogenic amino acid and endogenous alanine analogue precursor δ‐aminolaevulinic acid (5‐ALA), and the sensitivity of ESCs to dihydroartemisinin cytotoxicity was significantly increased (Fig 3B). We next assayed if different murine and human cancer cell lines respond similarly to the combinatorial treatment, independent of basal growth and sensitivity rates (Fig EV1G and H). We could confirm that all tested cancer cell lines gained hypersensitivity to dihydroartemisinin (Fig EV2A and B). Importantly, we also observed that the protoporphyrin inhibitor acifluorfen increased resistance of glioblastoma cells to dihydroartemisinin (Fig 3C) and reciprocally, 5‐ALA‐induced hypersensitivity in three independently derived human primary glioblastoma cell lines (Fig 3D; Fig EV2C and D).

Mechanistically, dihydroartemisinin has been shown to lead to the production of ROS (Witkowski & Halling, 1989; Antoine *et al*, 2014). Assessing ROS levels (using the redox‐sensitive fluorescent probe dihydroethidium, DHE) confirmed increased levels of ROS upon dihydroartemisinin treatment (Fig EV2E and F). We next assessed if 5‐ALA had any effect on ROS production in the absence or presence of dihydroartemisinin. While low concentrations of dihydroartemisinin and 5‐ALA alone had little or no effect on ROS levels or viability, the combination of both resulted in elevated ROS as well as a strong increase in cell death in human Jurkat T cells as well as human SHSY5Y neuroblastoma cells (Fig 3E and F; Fig EV2G and H). Similarly, and previously associated with dihydroartemisinin‐induced ROS induction (Antoine *et al*, 2014), mitochondrial polarization (ΔΨm, as assessed by the mitochondrial membrane potential probe JC‐1) was strongly elevated when cells were exposed to both dihydroartemisinin and 5‐ALA (Fig EV2I). Importantly, all of these phenotypes (ROS induction, mitochondrial depolarization, and cell death) could be suppressed by pharmacological inhibition of porphyrin production using a Ppox inhibitor (Fig 3E and F; Fig EV2G–I). These data indicate that modification of porphyrin biosynthesis is sufficient to modulate the sensitivity of multiple murine and human tumor cell lines to dihydroartemisinin toxicity.

### 5‐ALA and Artemisinin therapy in engineered human cerebral brain tumor organoids

Altered cell metabolism and upregulation of porphyrin production are frequently observed in human cancer cells (Navone *et al*, 1990). In human glioblastoma patients, elevated porphyrin biosynthesis is used diagnostically and therapeutically to localize and target tumor tissue, as porphyrin precursors (i.e., protoporphyrins) show strong fluorescence that can be monitored and functionally exploited *in vivo* (Batlle, 1993; Zhao *et al*, 2013; Hadjipanayis *et al*, 2015). Importantly, during fluorescence‐guided resection of brain tumors, preoperative oral administration of the clinically approved porphyrin enhancer 5‐ALA is used to distinguish high‐grade glioma from brain tissue, as fluorescent protoporphyrins, such as protoporphyrin IX, accumulate specifically in tumor cells but not the surrounding tissue, thus demonstrating selectively altered porphyrin biosynthesis in cancer (Stummer *et al*, 1998). Similarly to 5‐ALA, Artemisinin and its derivatives can readily pass through the blood brain barrier (de Vries & Dien, 1996). Since 5‐ALA is used to mark brain tumors for surgery, and the porphyrin pathway was found to be essential for Artemisinin toxicity, we wanted to apply our findings to treat therapy‐resistant brain cancer. We thus assessed if altered porphyrin biosynthesis in brain tumors can be therapeutically exploited, using a combination treatment of 5‐ALA and dihydroartemisinin.

**Figure 4:**
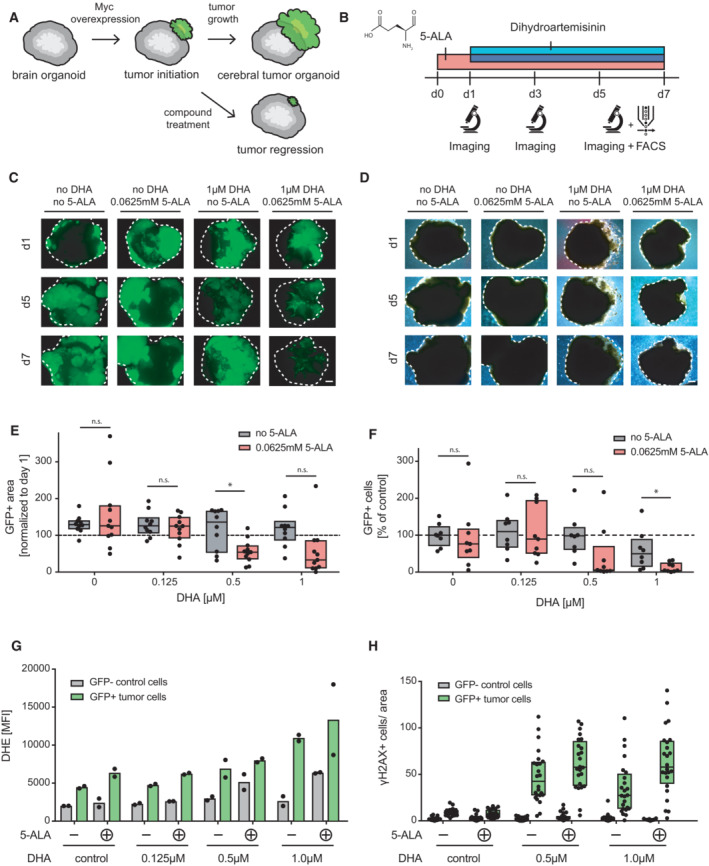
Cerebral tumor organoids (CNS‐PNET‐like) display increased sensitivity to dihydroartemisinin and 5‐ALA combination therapy A
Human cerebral tumor organoids for compound profiling.B
Schematic workflow of 5‐ALA‐ and DHA‐treated organoids.C, D
(C) Fluorescence and (D) brightfield images of DMSO (control), 5‐ALA (0.0625 mM), DHA (1 μM), or 5‐ALA and DHA (0.0625 mM and 1 μM) treated tumor organoids (CNS‐PNET‐like, c‐MYC overexpression). Organoids were imaged on day 1, day 5, and day 7. One experiment is shown, representative of four independent experiments. Scale bar 500 μm.E
Image analysis and quantification of GFP‐positive tumor areas of the treated organoids. At least eight organoids per condition were analyzed on day 3 and normalized to day 1. Three independent experiments were performed, with two to four organoids per batch, per condition. Data are shown as box plots (25^th^–75^th^ percentiles, median).F
Flow cytometry analysis of dissociated cerebral tumor organoids. Relative percentages of GFP^+^ tumor cells of normalized to control (DMSO) are shown on day 5. At least eight organoids per condition were analyzed. Three independent experiments were performed using all conditions, with two to four organoids each per batch per condition. Box plots (25^th^–75^th^ percentiles, median) of data are indicated.G
ROS/DHE staining and flow cytometry analysis of dissociated tumor organoids (PE, 582/15 nm). Mean fluorescence intensities (MFI) of DHE‐stained GFP^+^ tumor cells and GFP—control cells are indicated. *n* = 2, values are mean ± SD.H
Quantification of γH2AX^+^ cells in cerebral tumor organoids. 6 cryo‐sections of three organoids each were stained for γH2AX, scanned and 25 regions of interest (ROI 2,500 μm^2^) were analyzed per condition. Raw data were normalized to organoid area (per ROI) and are shown as box plots (25^th^–75^th^ percentiles, median). Data information: n.s., nonsignificant, *P < 0.05, unpaired Student's *t*‐test.

**Figure EV3:**
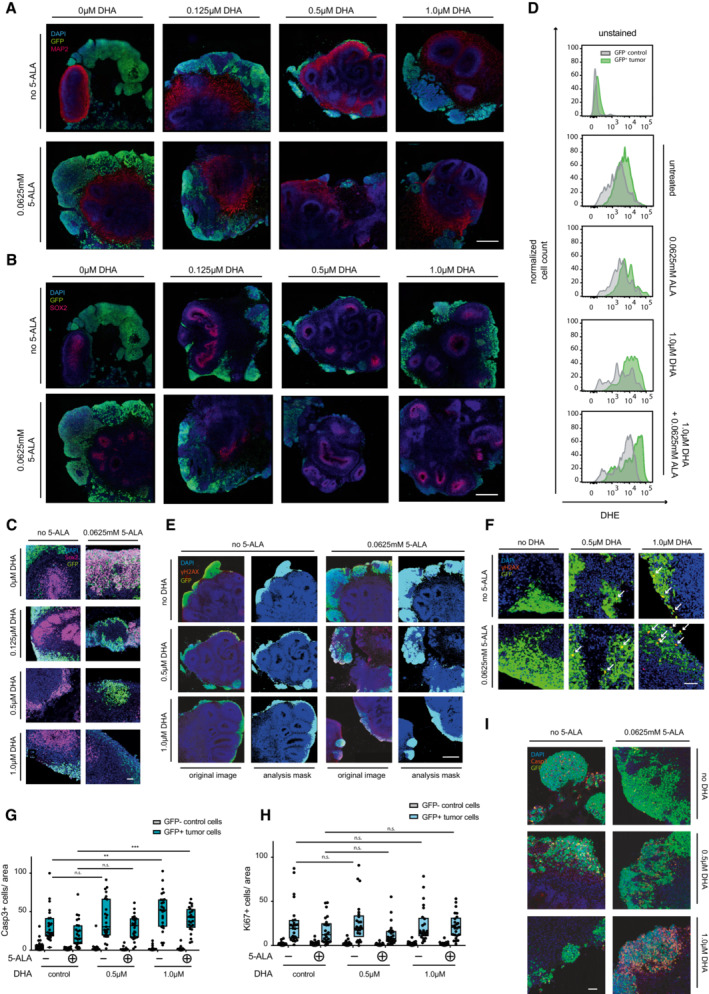
Dihydroartemisinin and 5‐ALA treatment of GFP^+^ tumor organoids induces ROS, DNA damage, and cell death A, B
Representative images of DAPI (blue), anti‐GFP (green), and (A) anti‐MAP2 (red) or (B) anti‐SOX2 (magenta) stained sections of control and DHA + 5‐ALA treated cerebral tumor organoids (central nervous system primitive neuroectodermal tumor (CNS‐PNET‐like) neoplasm model, c‐MYC overexpression). Scale bar 500 μm.C
Representative images of anti‐Sox2 (magenta), anti‐GFP (green), and DAPI (blue) stained sections of untreated and DHA and 5‐ALA‐treated organoids. Scale bar 50 μm.D
Representative FACS plots of ROS/DHE stained dissociated tumor organoids (CNS‐PNET‐like neoplasm, c‐MYC overexpression). Flow cytometry analyses (PE, 582/15 nm) of GFP^+^ tumor cells and GFP‐ wild‐type cells are shown. The experiment was repeated twice.E
Representative images and analysis masks of γH2AX, GFP, and DAPI stained and scanned tumor organoid slides. Scale bar 500 μm.F
Representative microscopy images of γH2AX, GFP, and DAPI stained tumor organoid sections. Arrows indicate γH2AX‐positive cells. Scale bar 50 μm.G, H
(G) Quantification of cleaved Caspase 3 (Casp3) and (H) Ki67‐positive cells in 5‐ALA‐ and DHA‐treated tumor organoids (CNS‐PNET‐like neoplasm). Per condition and group, 3 organoids, 6 sections each, were stained with antibodies to detect cleaved Caspase 3 or Ki67. Sections were imaged using a high‐magnification fluorescent scanner and 25 regions of interest (ROI, 2,500 μm^2^) were chosen and analyzed. The numbers of Casp3‐ or Ki67‐positive cells per GFP‐positive or GFP‐negative area were analyzed and are shown as box plots (median, 25^th^–75^th^ percentiles).I
Representative images of anti‐Casp3 (red), anti‐GFP (green), and DAPI (blue) stained sections of brain tumor organoids. Scale bar 50 μm. Data information: (G, H) Student's *t*‐test was used to analyze significance; n.s., nonsignificant, **P < 0.01, ***P < 0.001.

We used a recently established human cerebral tumor organoid model, which is based on genetic manipulation of neuronal precursors in the course of brain organoid development, thus recapitulating key aspects of human tumor formation under controlled *in vitro* conditions (Lancaster *et al*, 2013; Bian *et al*, 2018). Tumor cells in the organoids were simultaneously labeled with GFP, allowing monitoring of tumor growth over time and controlled compound profiling *in vitro* (Fig 4A). We first assessed tumor growth rates in the presence of dihydroartemisinin and/or 5‐ALA in a central nervous system primitive neuroectodermal tumor (CNS‐PNET‐like) neoplasm model, which is based on the overexpression of the c‐MYC oncogene (Bian *et al*, 2018; Fig EV2J). The highly malignant brain tumor organoids were treated with different doses of 5‐ALA, dihydroartemisinin, or a combination of both (Fig 4B). Since very high concentrations of 5‐ALA or dihydroartemisinin alone resulted in notable growth inhibition of GFP‐positive tumor organoids (Fig EV2J), concentrations were chosen for all subsequent experiments where the individual compounds had no notable effects on tumor or organoid growth. Strikingly, the combination of both 5‐ALA and dihydroartemisinin together markedly decreased GFP‐positive tumor cells in human cerebral organoids (Fig 4C and D; Fig EV2K). Quantification of the tumor tissue area at different time points (d3, d5) (Fig 4E; Fig EV2L) as well as FACS analysis (d5, d7) (Fig 4F) confirmed strong reduction of GFP‐positive cells upon 5‐ALA and dihydroartemisinin combination treatment. Immunohistochemistry and histology of sectioned brain organoids further showed that 5‐ALA treatment resulted in the loss of GFP‐positive tumor tissue, but had no apparent effect on nontransformed neuronal tissue in the cerebral organoids, such as rosette‐like structures and MAP2 or SOX2 expressing neuronal and progenitor cells (Figs EV2M and EV3A–C). Altogether, these data suggest that 5‐ALA and dihydroartemisinin combination strongly reduces the number of tumor cells in a human primitive neuroectodermal tumor (CNS‐PNET‐like) organoid model *in vitro*.

Since we observed a marked effect of 5‐ALA and dihydroartemisinin on ROS levels in cells, we performed DHE staining and analysis of the Myc overexpressing brain tumor organoids. Whereas GFP‐positive tumor cells displayed slightly higher basal ROS signals as compared to GFP‐negative wild‐type cells (as assessed by DHE staining and FACS analysis), ROS levels markedly increased upon 5‐ALA and dihydroartemisinin double treatment, especially in GFP‐positive cancer cells (Fig 4G; Fig EV3D). As elevated intracellular ROS also causes DNA damage (Cadet & Wagner, 2013), we assessed the amount of DNA double‐strand breaks (DSB) on organoid sections. We indeed observed a significant increase in γH2AX (phosphorylated histone H2AX)‐positive cells in 5‐ALA and dihydroartemisinin double‐treated GFP‐positive tumor tissue, but not in the control wild‐type cells (Fig 4H; Fig EV3E and F). Additionally, while the basal number of apoptotic (caspase 3‐positive) and proliferating cells (Ki67‐positive) was increased in GFP‐positive tumor cells as compared to normal tissue, apoptosis further increased in GFP‐positive tumor cells upon combination treatment with dihydroartemisinin and 5‐ALA (Fig EV3G–I).

**Figure 5:**
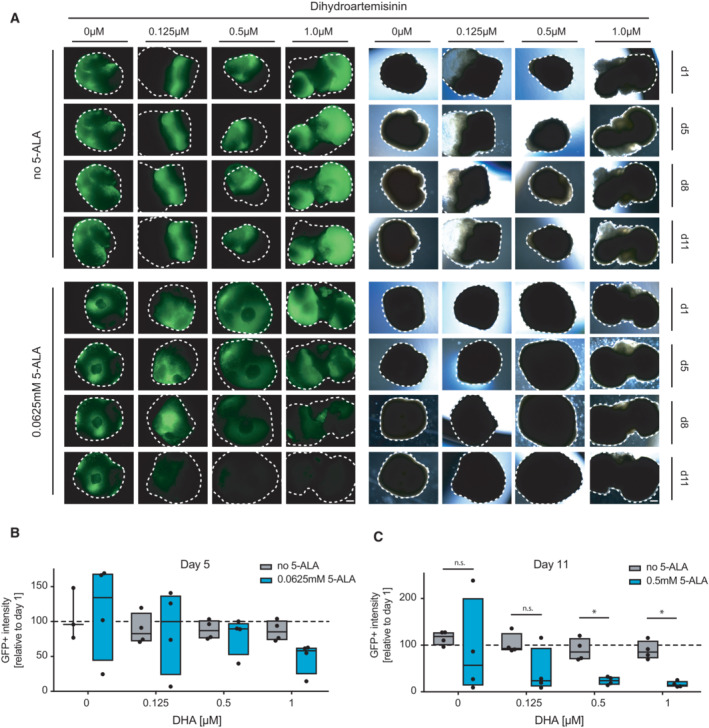
Dihydroartemisinin and 5‐ALA combination treatment of human glioblastoma‐like neoplastic organoids A
Fluorescence (left panel) and brightfield (right panel) images of 5‐ALA‐ and DHA‐treated human cerebral tumor organoids (glioblastoma‐like). Organoids were monitored on day 1, day 5, day 8, and day 11. One experiment is shown, representative of two independent experiments. Scale bar 500 μm.B, C
Image analysis and quantification of GFP‐positive tumor areas of the treated organoids. Four organoids per group were analyzed on day (B) d5 and (C) d11, normalized to day 1. Data are shown as box plots (25^th^–75^th^ percentiles, median) *n* = 4. Data information: n.s., nonsignificant, *P < 0.05, unpaired Student's *t*‐test.

**Figure EV4:**
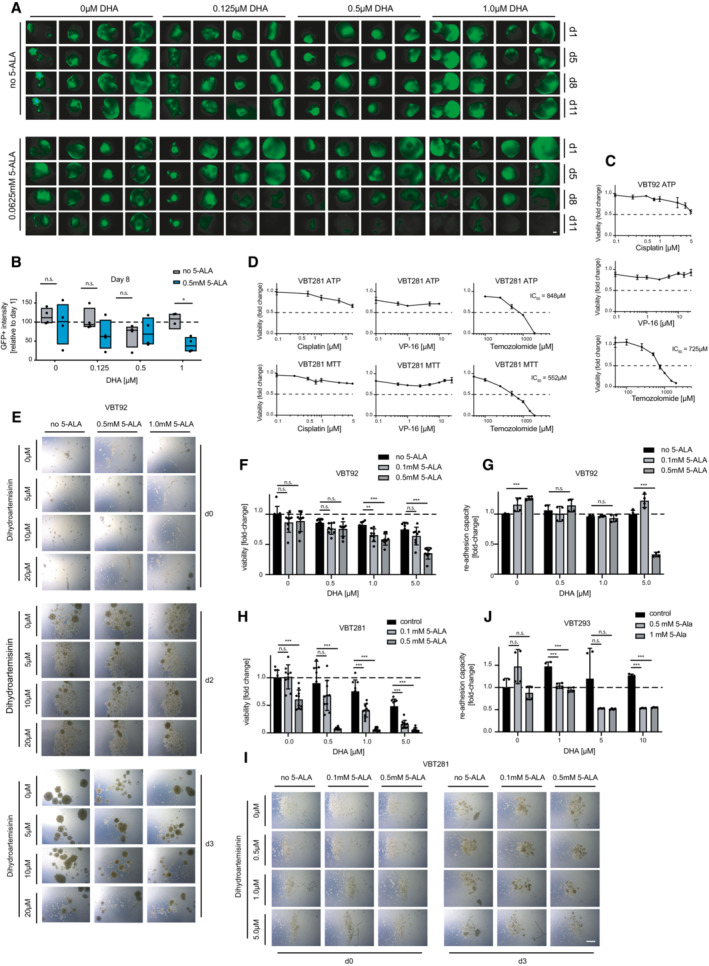
5‐ALA and dihydroartemisinin treatment of human glioblastoma‐like neoplastic organoids and patient‐derived glioma cells and spheroids A
Fluorescence images of 5‐ALA‐ and DHA‐treated cerebral tumor organoids (glioblastoma‐like neoplastic organoid model). Organoids were monitored on d1, d5, d8, and d11. Scale bar 500 μm.B
Quantification of GFP‐positive tumor areas of the treated brain tumor organoids. Organoids were analyzed on d8 and normalized to d1. Data are shown as box plots (25^th^–75^th^ percentiles, median). The experiment was performed in quadruplicate;C, D
Patient‐derived (C) high‐grade glioma (VBT92) and (D) atypical teratoid rhabdoid tumor cells (VBT281) were treated with the indicated doses of cisplatin (left), VP‐16 or etoposide (middle), and temozolomide (TMZ, right) for 72 h and their viability was assessed using (C, D) ATP‐based CellTiter‐Glo or (D) MTT/EZ4U assays. Experiments were performed in triplicate. Data are shown as mean ± SD.E
Representative brightfield images of 5‐ALA‐ and DHA‐treated patient‐derived high‐grade glioma spheroids (VBT92), taken on day 0, day 2, and day 3 of culture and treatment. Scale bar 500 μm.F
Viability of 5‐ALA‐ and DHA‐treated spheroids (VBT92) was assessed using the CellTiter‐Glo Luminescent Assay. Experiments were performed in quadruplicates. Data are shown as mean ± SD normalized to the untreated control.G
Readhesion capacity of replated spheroids (VBT92) as assessed by crystal violet staining. Experiments were performed in quadruplicates. Data are normalized to untreated control.H
Viability of 5‐ALA‐ and DHA‐treated patient‐derived atypical teratoid rhabdoid tumor (ATRT) spheroids (VBT281) was assessed using the CellTiter‐Glo Luminescent AssayPooled data of three independent experiments are shown, set up in quadruplicates or duplicates each. Data are shown as means ± SD.I
Representative images of 5‐ALA‐ and DHA‐treated spheroids (VBT281), taken on day 0 and day 3 of treatment are shown. Scale bar 500 μm.J
Quantification of the readhesion capacity of replated ATRT (VBT293) spheroids as assessed by crystal violet staining and absorbance measurement. Experiments were performed in quadruplicates. Data are shown as means ± SD. Data information: (B, F, G, H, J) n.s., nonsignificant, *P < 0.05, **P < 0.01, ***P < 0.001; Student's *t*‐test.

As 5‐ALA is routinely used clinically to visualize human high‐grade gliomas during fluorescence‐guided surgery, we next assessed 5‐ALA and dihydroartemisinin combination treatment in an *in vitro* human glioblastoma‐like neoplastic organoid model (Bian *et al*, 2018). These organoids were engineered to carry mutations of the tumor suppressor genes p53, NF1, and PTEN, together with GFP that allowed us to monitor tumor growth over time in the presence of dihydroartemisinin, 5‐ALA, or a combination of both. Whereas both 5‐ALA and dihydroartemisinin alone had little effect on the survival of untransformed as well as transformed cells, the combination of both compounds specifically ablated GFP‐positive tumor cells in the *in vitro* tumor model (Fig 5A; Fig EV4A). Quantification and image analysis of cerebral organoids confirmed progressive loss of GFP‐positive tumor cells and tissue, whereas untransformed cells were not affected (Fig 5B and C; Fig EV4B). These data show that 5‐ALA and dihydroartemisinin lead to increased cell death of tumor cells in two highly malignant brain tumor models, via elevated ROS levels and increased DNA damage.

**Figure 6:**
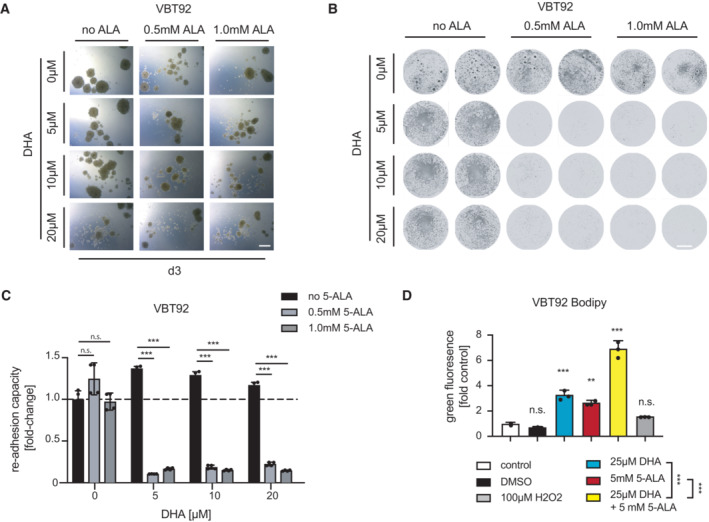
Human patient‐derived high‐grade glioma spheroids are highly susceptible to dihydroartemisinin and 5‐ALA combination therapy A
Representative brightfield images of 5‐ALA‐ and DHA‐treated patient‐derived glioblastoma spheroids (VBT92). Images were taken on day 3 of culture left untreated or exposed to the indicated treatments. Scale bar 500 μm. See also Fig EV4E.B
Replating of spheroids after 3 days of 5‐ALA and DHA treatment (duplicates). Representative brightfield images are shown for glioblastoma VBT92. Scale bar 500 μm.C
Quantification of the re‐adhesion capacity of re‐plated VBT92 spheroids as assessed by crystal violet staining and absorbance measurement. Data are shown as mean ± SD. Experiments were performed in quadruplicates.D
Lipid peroxidation in VBT92 cells treated with 5‐ALA and DHA. Lipid peroxidation was measured using BODIPY™ 581/591 C11 staining and green (from red) fluorescence shift was determined using flow cytometry. Experiments were performed in triplicate; values are shown as mean ± SD. Data information: n.s., nonsignificant, **P < 0.01, ***P < 0.001; (C) Student's *t*‐test (D) one‐way ANOVA followed by Bonferroni's multiple comparisons test.

### Treatment of drug‐resistant patient‐derived glioblastoma spheroids

**Figure EV5:**
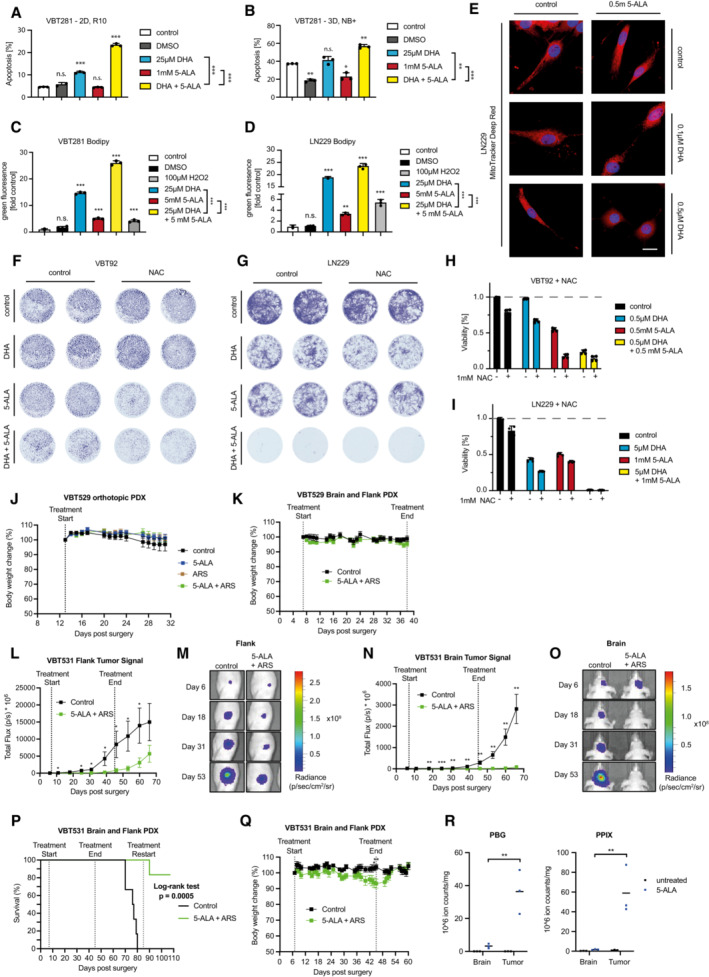
Mechanism of Artemisinin derivatives and 5‐ALA treatment of glioblastoma cells and PDX tumors in mice A, B
Apoptosis in 5‐ALA‐ and DHA‐treated glioblastoma cells (VBT281) grown (A) in a monolayer in 2D and (B) as spheroids in 3D. Apoptosis was measured using Annexin V and PI staining and quantified by flow cytometry. The number of apoptotic cells (double positive) as a percentage of total cells is shown. Experiments were performed in triplicate and values are shown as means ± SD, one‐way ANOVA followed by Bonferroni's multiple comparisons test.C, D
Lipid peroxidation in (C) VBT281 and (D) LN229 cells treated with 5‐ALA and DHA. Lipid peroxidation was measured using BODIPY 581/591 C11 staining and green (from red) fluorescence shifts were determined using flow cytometry. Experiments were performed in triplicate; values are shown as means ± SD, one‐way ANOVA followed by Bonferroni's multiple comparisons test.E
Representative images of mitochondrial structures in control and drug‐treated glioblastoma cells (LN229) using MitoTracker Deep Red staining. Scale bar 20 μm.F–I
NAC treatment of glioblastoma cells. (F, H) VBT92 and (G, I) LN229 cells were seeded and treated with DHA and 5‐ALA in presence and absence of the ROS scavenger NAC (1 mM, 1 h preincubation—clonogenic assay). Cells were stained with crystal violet and (F, G) imaged, and (H, I) absorbance was measured to determine relative viability. Experiments were performed in quadruplicate; values are shown as means ± SD.J
Body weight curves of orthotopic PDX mice treated with solvents (control), 5‐ALA, artesunate (ARS), or 5‐ALA plus ARS combined (same mice as in PDX main (B); *n* ≥ 5). K Body weight curves of mice injected with VBT529 glioblastoma cells into the flank and brain (same mice are shown as in PDX main (C–F); *n* ≥ 6).L, M
Quantification and representative pictures of the VBT531 flank tumor luminescence signal. 6–7 days after injection of 1.5 × 10^5^ VBT531 cells into the flank, mice received the following treatments: Control (solvents) or 5‐ALA plus ARS 4–5 times per week for 6 weeks via intraperitoneal injection (*n* = 6). After 2 weeks of treatment, concentrations of 5‐ALA (100 mg/kg) and ARS (50 mg/kg) were increased to 120 and 60 mg/kg, respectively.N, O
Quantification and representative pictures of VBT531 brain tumor luminescence signals. 6–7 days after stereotactic intracerebral injection of 1.5 × 10^5^ VBT531 cells, mice (*n* = 6) received treatments as described in (L and M).P
Kaplan–Meier survival curves of mice injected with VBT531 glioblastoma cells into their flanks and brains. Mice were treated with solvents (control) or 5‐ALA plus ARS (same mice as in J–O).Q
Body weight curves of mice injected with VBT531 glioblastoma cells into their flanks and brains (*n* = 6).R
Quantification of porphobilinogen (PBG) and protoporphyrin IX (PPIX) levels in the unaffected brain and tumor tissue in untreated versus 5‐ALA treated (120 mg/kg) glioblastoma‐bearing mice (*n* = 3). Data information: (J–R) Data are presented as means ± SEM; *P < 0.05, **P < 0.01, ***P < 0.001; (J, K, Q) two‐way ANOVA followed by Bonferroni's multiple comparisons test. (L, N, R) Student's one‐tailed, unpaired *t*‐test; (P) Log‐rank (Mantel‐Cox) test.

In addition to the mini‐brain model system, we next explored the effect of the compounds dihydroartemisinin and 5‐ALA in human patient‐derived high‐grade glioma neurospheres from children and adults who died of multidrug‐resistant brain cancer, cultured under stem cell conditions. As 5‐ALA is routinely used in the clinics to visualize human high‐grade gliomas during fluorescence‐guided surgery, porphyrin biosynthesis/metabolism in tumor cells differs from that in healthy brain tissue (Guyotat *et al*, 2016). The isolated cell models used for our therapeutic intervention experiments were classified as therapy‐resistant clinically by certified oncologists and based on their growth profiles in the presence of common cancer therapeutic drugs *in vitro* (Fig EV4C and D). Upon initiation of tumor sphere formation *in vitro*, treatment with 5‐ALA and dihydroartemisinin was started and growth of neurospheres as well as viability were monitored. 5‐ALA treatment of patient‐derived high‐grade glioma cells (VBT92) in the presence of dihydroartemisinin resulted in markedly reduced formation and expansion of spheroids (Fig 6A; Fig EV4E), as well as generally reduced viability (Fig EV4F). In addition, the readhesion capacity of spheroids was assessed and analyzed upon growth of pretreated cells in 3D cell cultures; this readhesion capacity was strongly reduced upon 5‐ALA and dihydroartemisinin combination treatment, but not by either compound alone (Fig 6B and C; Fig EV4G). These results could be confirmed in two additional patient‐derived spheroid models of atypical teratoid rhabdoid tumors (VBT281, VBT293), which also displayed high sensitivity to dihydroartemisinin and 5‐ALA as shown by reduced spheroid formation and cellular viability (Fig EV4H and I), as well as limited readhesion capacity (Fig EV4J). Moreover, when cytotoxicity was assessed in those patient‐derived brain cancer cells, we observed increased levels of apoptosis (PI, Annexin V) upon double treatment with 5‐ALA and dihydroartemisinin, both in 2D as well as 3D cultures (Fig EV5A and B). Mechanistically, these tumor cells also displayed higher levels of lipid peroxidation (Fig 6D; Fig EV5C and D) and a reduction of tubular mitochondrial structures upon combination treatment (Fig EV5E). However, addition of the ROS scavenger NAC (N‐acetyl‐l‐cysteine) did not apparently increase survival and did thus not salvage patient‐derived tumor cells from the combination of 5‐ALA and dihydroartemisinin (Fig EV5F–I). This data confirm strong 5‐ALA‐induced sensitization of human patient‐derived brain tumor cells to dihydroartemisinin in at least three independent *in vitro* model systems of different origin.

### Combined 5‐ALA and artesunate treatment reduce PDX glioblastoma growth in mice

**Figure 7:**
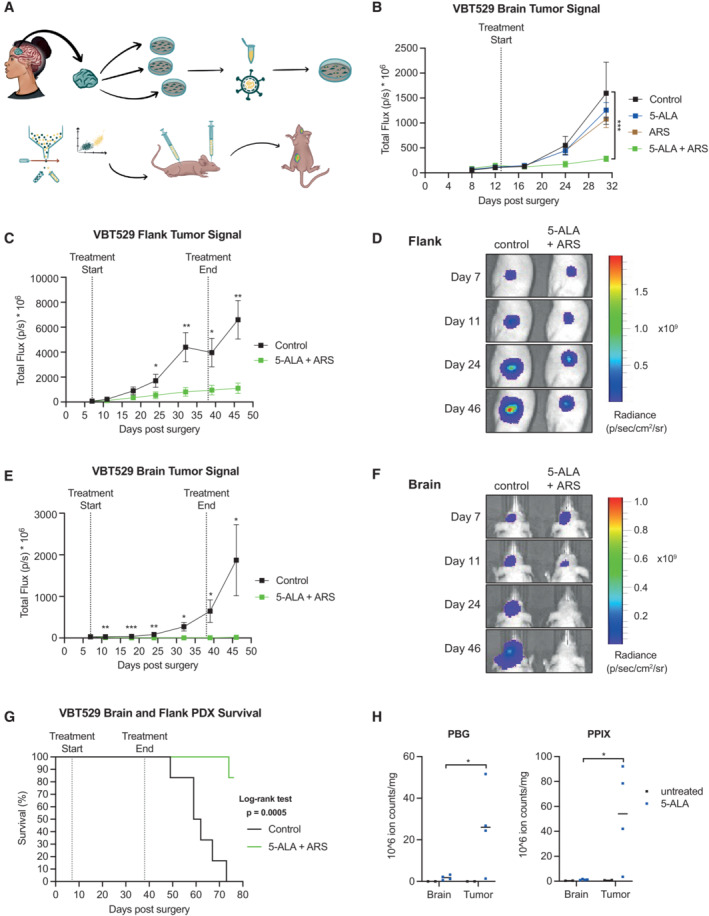
5‐ALA and artesunate treatment reduces glioblastoma growth in mice A
Schematic outline of flank and brain PDX models. Patient‐derived tumor cells (VBT529) were cultured *in vitro* and transduced with a Luc2‐iRFP lentiviral vector. Following flow cytometry sorting, LUC2 expressing cells were injected into the brain or flank and brain of *nude* mice. Upon tumor development, mice were treated with the respective compound(s) and tumor growth was monitored using the IVIS *in vivo* imaging system.B
*In vivo* efficacy comparison of solvents (control), 5‐ALA, artesunate (ARS) and combined treatment in an orthotopic glioblastoma model. 11–14 days after stereotactic intracerebral injection of 2 × 10^5^ VBT529 cells, mice received the following treatments: Control (solvents), 5‐ALA (80 mg/kg), ARS (40 mg/kg), or 5‐ALA plus ARS 5 times per week via intraperitoneal injection (*n* ≥ 5).C, D
Quantification and representative images of the VBT529 flank tumor luminescence signal. 6–7 days after injection of 1.5 × 10^5^ VBT529 cells into the flank, mice received the following treatments: Control (solvents) or 5‐ALA plus ARS 4–5 times per week for 5 weeks via intraperitoneal injection (*n* = 6). After 4 days of treatment, concentrations of 5‐ALA (100 mg/kg) and ARS (50 mg/kg) were increased to 120 and 60 mg/kg, respectively. The 20% dosage increase of both drugs was used as the body weight, behavior, and overall health of the mice were not affected by both, the lower as well as higher dosages.E, F
Quantification and representative images of VBT529 brain tumor luminescence signals. 6–7 days after stereotactic intracerebral injection of 1.5 × 10^5^ VBT529 cells, mice received the treatments as described in (C and D) (*n* = 6).G
Kaplan–Meier survival curves of mice injected with VBT529 glioblastoma cells into the flank and brain (same mice as in C–F; *n* = 6). Mice were treated with solvents (control) or 5‐ALA plus ARS (same mice as in C–F).H
Quantification of porphobilinogen (PBG) and protoporphyrin IX (PPIX) levels in untreated versus 5‐ALA treated (120 mg/kg) glioblastoma‐bearing mice (*n* ≥ 2). Levels in tumors as compared to surrounding brain tissue are shown. Data information: (B, C, E) Data are presented as mean ± SEM; *P < 0.05, **P < 0.01, ***P < 0.001; (B) two‐way ANOVA followed by Bonferroni's multiple comparisons test; (C, E, H) Student's one‐tailed, unpaired *t*‐test, or (G) Log‐rank (Mantel–Cox) test.

To assess whether our combinatorial treatment exhibits synergistic antitumor activities *in vivo*, we established patient‐derived xenografts (PDX) models from two subpopulations (VBT529 and VBT531) of a surgically removed, aggressive glioblastoma. Both lines were tagged with Luc2‐iRFP before being injected into nude mice (Fig 7A). In an orthotopic setup where human glioblastoma cells were injected into the brain, mice were treated with 5‐ALA (80 mg/kg), artesunate (ARS, 40 mg/kg), or the double combination, starting 11–14 days post‐injection. Artesunate is an Artemisinin derivative that shows high brain penetrance is rapidly metabolized to dihydroartemisinin *in vivo* and is commonly used in patients and animal studies (Navaratnam *et al*, 2000). Five intraperitoneal treatments with the double combination per week resulted in a significant reduction of tumor growth as compared to the solvent control as well as to both single treatments (Fig 7B), confirming the strong synergistic antitumor activity from the *in vitro* studies. Importantly, neither treatment regimen showed noticeable effects on mouse body weight, behavior, or overall well‐being (Fig EV5J).

We next asked whether drug concentrations can be increased to boost the antineoplastic effect and whether the tumor location affects treatment efficacy. Therefore, VBT529 cells were injected both into the mouse brain and flank. 6–7 days post‐injection, mice were treated with the double combination (up to 120 mg/kg 5‐ALA and 60 mg/kg ARS) or the control solvent 4–5 times per week for 1 month. While control mice developed malignant tumors in the brain and the flank, the combinatorial treatment significantly reduced tumor growth in both locations (Fig 7C–F). Intriguingly, antitumor activity was most striking in the brain, where basically no tumor progression was observed (Fig 7E and F). Mouse body weights remained unaffected in these therapeutic scenarios (Fig EV5K). The strong reduction in tumor growth manifested in a significant increase in overall survival of treated mice, which was monitored until day 76 post‐injection (Fig 7G).

To elucidate why the combinatorial treatment specifically targets tumor tissue, we measured two heme pathway metabolites in brain and tumor tissue 60 min post‐intraperitoneal injection of 120 mg/kg 5‐ALA in control group mice. Interestingly, both assessed metabolites, porphobilinogen and protoporphyrinogen IX, showed higher levels in brain tumor as compared to the surrounding normal brain tissue upon 5‐ALA treatment, indicating a higher activity of the heme biosynthesis pathway in these cells (Fig 7H). Importantly, all results obtained with the VBT529 cell line could be replicated with the second tumor line VBT531 (Fig EV5L–R). Collectively, these data demonstrate a strong synergistic activity of 5‐ALA and ARS against human relevant glioblastoma models *in vivo*.

## Discussion

In this work, we delineate porphyrin biosynthesis as the critical endogenous pathway for Artemisinin‐triggered cytotoxicity in eukaryotic cells. Using different high‐throughput genetic screening systems in two unique eukaryotic model organisms, we have independently identified mitochondrial function, and more specifically porphyrin/heme biosynthesis, to be required for the cytotoxic activity of the antimalarial and anticancer compound. Genetic as well as pharmacological modulation of porphyrin production was sufficient to modulate dihydroartemisinin toxicity, as well as to control the levels of dihydroartemisinin‐induced ROS, in multiple different cellular contexts and species. Furthermore, we could show in several independent *in vitro* human cerebral tumor organoid, drug‐resistant spheroid, and orthotopic PDX glioblastoma model systems that we can specifically target brain tumor cells using a combination treatment of 5‐ALA and dihydroartemisinin to induce cell death.

Artemisinin and its derivatives have been widely used for the treatment of malarial and parasitic worm (helminth) infections, but in China they are also used for the therapy of systemic lupus erythematosus and liver cancer due to their cytotoxic activity at higher doses (Bessone *et al*, 2014). The exact mechanism of action of Artemisinin was not known because of the complex chemical interactions involved (Haynes *et al*, 2013). For malaria, the endoperoxide ring can be activated by heme inside the hemoglobin of the red blood cells, resulting in the production of free radicals that in turn damage susceptible proteins, resulting in the death of the parasite (Tilley *et al*, 2016). However, unlike other antimalarials that kill the parasite at a particular stage of its life cycle, Artemisinin is able to kill at all the life cycle stages (Golenser *et al*, 2006), suggesting additional targets. Moreover, it has been reported that Artemisinin can bind to a plethora of additional proteins (Zhang *et al*, 2015), further indicating that it might act on multiple cellular targets critical for its anti‐inflammatory and cytotoxic activity. Our data in yeast HIP‐HOP as well as murine haploid ESC screens now unequivocally and surprisingly identify a singular pathway that controls Artemisinin cytotoxicity, namely porphyrin/heme biosynthesis. In fact, while we cannot formally exclude other pathways, we identified every single enzyme involved in porphyrin/heme biosynthesis with very high significance scores, including the generation of metabolites that critically feed into this pathway. This allowed us to use the rate‐limiting metabolite 5‐ALA, already routinely used by neurosurgeons to intraoperatively visualize brain tumors (Zhao *et al*, 2013), to enhance porphyrin biosynthesis and thereby sensitize tumor cells to the cytotoxic effects of dihydroartemisinin. Intriguingly, this sensitization could be observed in essentially all tumor cell lines we tested and could be blocked by the Ppox inhibitor acifluorfen, again providing evidence that dihydroartemisinin cytotoxicity is critically dependent on the final step of porphyrin biosynthesis. Importantly, these data are in line with our finding that dihydroartemisinin/5‐ALA treatment markedly induces ROS and thereby damages DNA as determined by γH2AX and JC‐1 staining, providing a mechanistic explanation how and why Artemisinin can kill multiple types of tumor cells.

Brain tumors remain one of the deadliest neoplasms with often limited treatment options. For instance, glioblastoma is one of the most aggressive types of brain cancer, representing about 15% of all brain tumors (Young *et al*, 2015). Treatment usually involves surgery, radiation therapy, and chemotherapy with temozolomide (De Vleeschouwer *et al*, 2017). Unfortunately, despite radical multimodal treatments, glioblastomas often recur, resulting in an overall survival of approximately 15 months and 3–7% of people diagnosed with glioblastoma survive longer than 5 years (Bi & Beroukhim, 2014; Ostrom *et al*, 2016). A current clinical trial protocol is using eight different types of drugs to treat recurring glioblastoma (Parasramka *et al*, 2017). Thus, it is paramount to find novel treatment options, one of which is innovative immunotherapy such as tumor vaccines or CAR T cells (Jacob *et al*, 2020). Recent progress in the engineering of cerebral organoids from stem cells has allowed to model and experimentally dissect human brain tumor development, including glioblastomas (Bian *et al*, 2018). Using these two different models (CNS‐PNET‐like and glioblastoma‐like neoplasm) of inducible cerebral brain tumor organoids, we were indeed able to show that a combination of 5‐ALA and dihydroartemisinin selectively and efficiently kills tumor cells, while not affecting the surrounding “normal” neuronal tissue of the organoids. To further explore the clinical efficacy and recapitulate parental tumor heterogeneity, we also tested whether 5‐ALA and dihydroartemisinin is cytotoxic in patient‐derived brain tumor spheroids. Intriguingly, in three different patient‐derived organoid models, all of which were defined as multidrug resistant in the clinic and in *in vitro* cell assays, the addition of 5‐ALA markedly sensitized the tumor cells to Artemisinin killing. Most importantly, in patient‐derived xenograft models of human glioblastoma, the combination treatment of 5‐ALA and the Artemisinin derivative artesunate almost entirely abolished tumor cell growth in mice *in vivo*. Notably, a recent study has also reported enhanced killing of a colon cancer cell line by 5‐ALA and Artemisinin (Wang *et al*, 2017), supporting our results in engineered brain cancer organoids, patient‐derived brain tumor spheroids and orthotopic PDX glioblastoma models.

In conclusion, our HIP‐HOP and haploid ESC screens have identified the critical pathway of how Artemisinin kills eukaryotic cells, a pathway that appears to be conserved from yeast to human. Metabolic perturbation of this pathway, heme/porphyrin biosynthesis, can be used to abolish or sensitize tumor cells to the cytotoxic effects of Artemisinin. Since both 5‐ALA and Artemisinin, as well as its derivates, have been safety tested for brain tumor surgery and literally millions of malaria patients, our drug combination could be readily translated to treat patients with yet untreatable and multidrug‐resistant brain tumors.

## Materials and Methods

### HIP‐HOP assays

The growth‐inhibitory potency of compounds was determined using wild‐type *S. cerevisiae* BY4743 as published in detail (Hoepfner *et al*, 2014). HIP‐HOP analysis was performed as described previously (Pierce *et al*, 2006; Hoepfner *et al*, 2014). Sensitivity was computed as the median absolute deviation logarithmic (MADL) score for each compound (Dihydroartemisinin, DHA)/concentration (HIP—3 μM, HOP—10 μM) combination. Z‐scores were based on a robust parametric estimation of gene variability from > 4,000 different profiles and computed as described in detail previously. In brief, IC (inhibitory concentration) scores were determined using serial dilutions with log phase yeast cultures in YPD (2% glucose, 2% BactoPeptone, 1% yeast extract) in 96‐well plates (starting OD_600_ 0.05, 120 μl/well) at 30°C, 770 rpm for 16 h. IC_10_, IC_20_, IC_30_, IC_40_, and IC_50_ concentrations were determined for all compounds. The HIP assays were performed in 24‐well plates (1,600 μl/well YPD) at IC_30_ concentrations in duplicate. YPD/compound filled wells were inoculated with ∼ 250 yeast cells/strain (1.5 OD_600_/ml o/n culture), plates were incubated for 16 h at 30°C, 550 rpm for 5 doublings each, for a total of 20 generations. Similarly, for HOP assays, cultures were incubated with the compounds and 5 doublings were monitored (no further dilutions). For each HIP/HOP experiment, growth curves were recorded and analyzed. gDNA isolation, TAG PCR amplification, GenFlex Tag16K v2 hybridization, and data analysis have been described previously (Hoepfner *et al*, 2014).

### Yeast growth rates

Growth assessments and MIC determinations were performed by filling plates with YPD (2% glucose, 2% BactoPeptone, 1% yeast extract) or YPEG (2% glycerol, 1% ethanol, 2% BactoPeptone, 1% yeast extract) and solidified using 2% agar. Compounds were diluted in DMSO and added on top of the plates, and DMSO concentrations were kept constant at 2%.

### Mitotracker experiments

LN229 cells were seeded in 8‐chamber slides (chambered coverslip with 8 wells, ibidi GmbH) at a density of 3 × 10^4^ cells per ml in 300 μl Dulbecco's Modified Eagle's Medium with 10% FBS per well. The chamber slide was incubated under normal conditions (37°C and 5% CO2). After 24 h, cells were exposed to DHA and/or 5‐ALA for a period of 24 h. Then, medium was aspirated, and 300 μl of medium without FBS and phenol red were added, as well as 50 nM of Mitotracker (Mitotracker red, CMXRos, M7512 Invitrogen, Thermo Fisher Scientific) for 30 min. Slides were fixed with 4% PFA for 20 min at room temperature, washed with 1xPBS, and covered with Vectashield (Vectashield with DAPI, Vector Laboratories Inc.). Slides were kept at 4°C and then cells were imaged on a laser scanning microscope (Carl Zeiss), with a 63× oil immersion objective and using the respective image software (Zen2010, Carl Zeiss). Signals were detected using 405 and 555 nm solid state laser lines and 555 nm short pass (DAPI) and 549 nm long pass (A549) emission filters, respectively.

### Mitochondrial staining

MitoTracker staining (Cat. No. M7514, ThermoFisher Scientific) was performed according to the manufacturer's protocol and imaged on an Axiovert 200M with standard FITC filter sets, through a 100×/1.3 Ph3 objective (Carl Zeiss, Oberkochen, Germany) using a X‐Cite120 (Excelitas, Waltham, Massachusetts, USA) illumination light source.

### Mammalian cell lines

Mouse AN3‐12 ESC lines were generated in our laboratory and characterized and authenticated as previously described (Elling *et al*, 2011). Haploid murine ESCs were used for insertional mutagenesis and derivation of gene trap knockout cell lines. AN3‐12 cells (Elling *et al*, 2011) were cultured in DMEM supplemented with 15% fetal bovine serum (FCS), penicillin–streptomycin, nonessential amino acids (NEAA), sodium pyruvate (1 mM), l‐glutamine (2 mM), β‐mercaptoethanol (0.1 mM), and LIF (20 μg/ml). SH‐SY5Y were obtained directly from the supplier (Sigma Aldrich) and used for growth assays and cellular stainings. SH‐SY5Y cells were cultured in DMEM/F12 1:1 supplemented with 10% FCS (fetal calf serum), penicillin–streptomycin, and L‐glutamine. 4T1 cells were cultured in IMDM supplemented with 10% fetal calf serum (FCS), penicillin–streptomycin and L‐glutamine. Jurkat T cells were used in *in vitro* viability and DHE assays; the cells were obtained from an in‐house source and are functionally described elsewhere (Reikerstorfer *et al*, 1995). Mcf7, MDA‐MB‐231, 4T1, Panc1, and B16F10 cancer cell lines were obtained in house. MEFs (mouse embryonic fibroblasts) were generated and obtained in house. PlatE cells were used for recombinant retrovirus and lentivirus production as previously described (Stadlmann *et al*, 2017; Taubenschmid *et al*, 2017). MEFs, Mcf7, MDA‐MB‐231, Panc1, and B16F10 and PlatE cells were cultured in DMEM supplemented with 10% FCS, penicillin–streptomycin and L‐glutamine. Cells were cultured at 37°C, with 20% O_2_ and 5% CO_2_. All cell lines tested were negative for mycoplasma. No cell lines listed by ICLAC were used.

### Competitive growth assays

Haploid ESCs (Elling *et al*, 2011) harboring gene traps in genomic introns were seeded at low density in normal ESC growth medium and infected for 12 h with two viruses, one encoding mCherry plus Cre recombinase and the other coding for GFP, both expressing puromycin (Invivogen, ant‐pr‐1). Infected cells were selected (final concentration of puromycin, 1 μg/ml) after 24 h and expanded. Ratios of GFP‐ to mCherry/Cre‐expressing cells in the presence and absence of dihydroartemisinin were determined using high‐throughput flow cytometry (BD LSRFortessa HTS Cell Analyzer).

### Compound profiling in cell lines

The following compounds were used: Dihydroartemisinin (dihydroqinghaosu, artenimol, DHA) was a kind gift from Dominic Hoepfner (Novartis); Delta‐aminolaevulinic acid hydrochloride, 5‐ALA (Sigma Aldrich A3785‐500MG); acifluorfen, protoporphyrinogen oxidase (Ppox) inhibitor (Sigma Aldrich N11027‐250MG); piperlongumine, induces ROS (Sigma Aldrich SML0221‐5MG). For dosage responses, cells were seeded in 96‐wells (25,000/96‐well, in triplicate—where indicated) and subjected to compounds for 48 h (or 72 h if indicated in the Figure). Cell viability was assessed using automated cell counting (high‐throughput flow cytometry), Alamar Blue staining (Invitrogen, DAL1100) or CellTiter‐Glo Luminescent Assay (Promega, G7570, according to the manufacturer's protocol), respectively. For the detection of ROS, treated cells were collected, washed with 1x HBSS (without Ca^2+^ and Mg^2+^), incubated with 1 mM DHE (dihydroethidium (hydroethidine), Invitrogen, D11347) in 1× HBSS for 45 min at 37°C, washed twice and counterstained with DAPI or a viability dye (eBioscience™ Fixable Viability Dye eFluor™ 780, 65‐0865‐18) for 10 min on ice. Cells were then collected, strained, and analyzed using flow cytometry for DHE in the red fluorescent spectrum (PE channel 582/15 nm). For JC‐1 staining, treated cells were collected, washed with 1xPBS, incubated with 2 μM JC‐1 in 1xPBS (MitoProbe JC‐1 Assay Kit‐1, Invitrogen, M34152) for 35 min at 37°C, washed twice, and analyzed using flow cytometry in the red (PE channel) and green (FITC channel) fluorescence spectrum.

### Cerebral organoids

Cerebral organoids were generated as described (Lancaster *et al*, 2013). Human ESCs (feeder‐free H9, WiCell) were transferred into low‐attachment 96‐well‐plates (Corning) in a density of 9,000 cells per well and incubated in human stem cell medium. After 6 days, the medium was changed to neural induction media, containing Dulbecco's modified eagle medium DMEM/F12, N2 supplement (Invitrogen), Glutamax (Invitrogen), minimum essential media‐nonessential amino acids (MEM‐NEAA), and 1 μg/ml heparin (Sigma), to promote growth of ectodermal tissue. On day 11, embryonic bodies (EBs) were embedded in droplets of Matrigel and transferred to differentiation medium, containing DMEM/F12: Neurobasal 1:1, N2 supplement (Invitrogen), B27 supplement (without vitamin A) (Invitrogen), 50 μM 2‐mercaptoethanol, 1:4,000 insulin (Sigma), Glutamax (Invitrogen), penicillin–streptomycin, MEM‐NEAA onto 10 cm plates. Five days later, organoids were transferred to an orbital shaker and maintained in differentiation media containing vitamin A (in B27 supplement). Human cells were tested negative for mycoplasma.

### Cerebral tumor organoid formation

Tumor initiation was induced by either oncogene amplification, using a Sleeping Beauty (SB) transposase, or mutation of tumor suppressor genes, using the CRISPR‐Cas9 system in day 10 old embryoid bodies (EBs). Plasmids carrying the transposase as well as GFP and the desired oncogenes or expressing Cas9 nuclease were introduced by electroporation. In brief, a mixture of 1 μg DNA and 100 μl nucleofector solution was added to 10 EBs and transferred to a nucleofection cuvette. The Lonza Nucleofector 2b and the A‐023 program were used for nucleofection. Thereafter, EBs were transferred into 10 cm dishes comprising of differentiation media plus vitamin A and embedded into Matrigel 24 h later. Two different types of tumors were initiated (Bian *et al*, 2018): Central nervous system primitive neuroectodermal tumors (CNS‐PNET‐like) were induced by the overexpression of c‐MYC. Glioblastoma‐like tumor group 2 (GBM‐2) was modeled by mutagenesis of the tumor suppressor genes p53, NF1, and PTEN. All plasmids were designed by Shan Bian at IMBA (Bian *et al*, 2018).

### Compound profiling in cerebral tumor organoids

Cerebral tumor organoids were treated with different doses of dihydroartemisinin and 5‐ALA, and the survival and growth of transformed and untransformed neurons were monitored. Fluorescence labeling of tumor cells allowed us to distinguish transformed GFP^+^ cells from nontransformed cells in the same organoid throughout the time of experiment using brightfield microscopy combined with fluorescence imaging. At the endpoint of the treatment (d5 or d7), cerebral (tumor) organoids were enzymatically and mechanically dissociated using 1× trypsin, 35–45 min incubation at 37°C, and gentle shaking. Organoid cells were singularized by careful resuspension, addition of differentiation medium and straining (Falcon Round‐Bottom Tubes with Cell Strainer Cap, 5 mL, 35 μm nylon mesh cell strainer snap cap). Single cells in suspension were counterstained with DAPI or the viability dye (eBioscience™ Fixable Viability Dye eFluor™ 780, 65‐0865‐18), and the amounts of GFP‐positive cells were analyzed by flow cytometry (BD LSRFortessa HTS Cell Analyzer). 5‐ALA‐ or dihydroartemisinin‐treated cerebral organoids were imaged at the indicated time points using the Axio Vert.A1 Inverted Microscope system (Zeiss Objective EC Plan‐Neofluar 2.5×/0.085 Pol M27, 0.5 camera adapter). Brightfield and green fluorescence images were taken from the same area. Additionally, for image analyses, the Celldiscoverer 7 (Zeiss), a fully integrated high‐end automated live cell imaging system, was used.

### Immunohistochemistry staining

Cerebral organoids were fixed in 4% PFA (room temperature, 1 h), incubated with 30% sucrose (4°C, o/n), OCT (Tissue‐tek OCT Compound, SANOVA PHARMA GESMBH, 4583) embedded and 20 μm sections were cryostat cut (−12/−14°C). For staining, sections were blocked and permeabilized for 1 h on RT in 0.25% Triton X‐100, 4% donkey serum in PBS, stained with primary antibodies diluted in 0.1% Triton X‐100, 4% donkey serum in PBS at RT overnight, incubated with secondary antibodies diluted in 0.1% Triton X‐100, 4% donkey serum in PBS for 2 h at RT, and finally counterstained with DAPI (4′,6‐diamidino‐2‐phenylindole, Dilactate, Invitrogen, D3571) at RT for 20 min. Fluorescence Mounting Medium (Dako, S302380) was used for mounting the sample slides. The following antibodies were used: anti‐GFP antibody (Abcam, ab13970, 1:500), anti‐Ki‐67 monoclonal antibody (SolA15, eBioscience, 14‐5698‐82, 1:100), anti‐cleaved caspase‐3 antibody (Asp175, Cell Signaling Technology, 966S1, 1:300), anti‐Sox2 antibody (R&D Systems, AF2018; or Abcam, ab97959, 1:1000), anti‐Map2 antibody (Millipore, MAB3418, 1:500), and anti‐gamma‐H2A.X antibody (phospho S139, Abcam, ab11174, 1:300). The organoids were imaged on an LSM780 Axio Imager (point laser scanning confocal microscope, GaAsP (gallium arsenide) detectors with QE of 45% and up to 2× SNR) with a standard filter set (CH1: 371–735, CH2: 479–735, CH3 Quasar (GaAsP): 416–690) through a 20×/0.8 plan‐Apochromat objective (Carl Zeiss) using laser illumination (laser diode 405—25 mW, argon 458, 488, 514—30 mW, DPSS 561—15 mW, HeNe 633—5 mW).

### Patient‐derived brain tumor models

The patient‐derived brain tumor models VBT12, VBT92, VBT281, VBT293, VBT529, and VBT531 were established at the Medical University of Vienna, Austria, and the models BTL53, BTL90, and BTL1333 at the Johannes Kepler University Hospital, Linz, Austria, as previously published (Gabler *et al*, 2019). The study was approved by the local ethics committee. Patient‐derived tumor cells were cultivated in RPMI‐1640 (R6504, Sigma‐Aldrich) supplemented with 10% fetal bovine serum (FBS, 11403164, Thermo Fisher Scientific) and grown at 37°C and 5% CO_2_. Neurospheres were cultured in low attachment plates in neurobasal media (NB^+^, Neurobasal Media, Gibco, 21103‐049, Thermo Fisher Scientific) supplemented with 1% B27 (50×, 175404‐044, Thermo Fisher Scientific), 1% N2 (Neuro Mix 100×, 17502‐048, Thermo Fisher Scientific), 20 ng/ml basic FGF (100‐18B, PeproTech), 20 ng/ml EGF (E9644, Sigma‐Aldrich), and 2 mM L‐glutamine (Sigma Aldrich). Monitoring of cells and imaging was performed with a Zeiss Primo Vert light microscope (Carl Zeiss, Jena, Germany). All cell lines were tested negative for mycoplasma.

### Cell viability assays

Cells were seeded in triplicates in 96‐well plates (CytoOne^®^ Starlab GmbH) at a density of 2.5 (VBT92) or 4 (VBT281) × 10^4^ per ml of RPMI‐1640 based culture media using 100 μl per well and, after 24 h of adherence, treated with the indicated doses of cisplatin (Sigma Aldrich, 232120‐50MG), VP‐16 or etoposide (Sigma Aldrich, E1383‐25MG), or temozolomide (TMZ, Sigma Aldrich, T2577‐100MG) for 72 h at 37°C, 5% CO2. The number of viable cells was determined using “CellTiter‐Glo^®^ Luminescent Cell Viability Assay” (Promega G7571) or MTT assay, according to the manufacturer's instructions (MTT/ EZ4U assay, Biomedica BI‐5000).

For spheroid assays cells were seeded at a density of 1 × 10^4^ cells per well in 500 μl neurobasal media (NB^+^ with supplements, see above) in duplicates in ultralow attachment 24‐well plates (Corning). Prior to drug exposure, in both settings, cells were left for 24 h under normal culture conditions. Next, the indicated concentrations of DHA and/or 5‐ALA were added either in RPMI 1640 with 10% FBS or neurobasal media. Following drug exposure of 72 h, cell viability was assessed using “CellTiter‐Glo^®^ Luminescent Cell Viability Assay” (Promega G7571) according to manufacturer's protocols. Luminescence was measured at 1000 nm at the Tecan infinite 200Pro (Zurich, Switzerland). Raw data were analyzed using GraphPad Prism software 8.0.1 (GraphPad Software). Results are given as mean ± standard deviation (SD) and were normalized to untreated control cells.

### Re‐adhesion capacity of patient‐derived brain tumor spheroids

Cells were seeded at a density of 1 × 10^4^ cells per well in 500 μl neurobasal media in duplicates in ultralow attachment 24‐well plates at 37°C and 5% CO_2_, 24 h prior to drug treatment. Following compound exposure for 72 h, cells were harvested (280 *g*, 5 min), resuspended in growth media (RPMI‐1640, 10% FBS), and cultured in 24‐well plates for 7 days to allow and follow attachment. Afterward, the medium was discarded, plates were dried upside‐down overnight, and cells were fixed in ice‐cold methanol for 15 min at 4°C before crystal violet staining. Images were taken using a Nikon D3200 camera and processed using ImageJ software (ImageJ Fiji). For quantification, crystal violet was eluted using 2% sodium dodecyl sulfate, and color absorbance was measured at 560 nm at the Tecan infinite 200Pro. Analysis was performed in quadruplicates. Values were analyzed using GraphPad Prism software 8.0.1 and are given as arbitrary units (AU) as mean ± SD normalized to untreated control.

### Annexin V‐PI staining

VBT281 glioblastoma cells were seeded in 6‐well plates (CytoOne) for adherent cell experiments, or ultra‐low attachment 6‐well plates (Corning) for spheroid assays, at a cell density of 1 × 10^5^ cells per ml of the respective cell culture media. R10 media (RPMI with 10% FBS) was used for adherent cells. NB^+^ media (Neurobasal media with supplements) was used for spheroid cultures. After preplating for 24 h, cells were treated with DHA (dihydroartemisinin), 5‐ALA, or the combination at the indicated concentrations.

The supernatant of adherent cells was combined with the trypsinized, singularized cells, washed with 1× PBS and filtered through a nylon mesh (size 60 μm, Sigma‐Aldrich). Floating spheroids were collected, washed with 1× PBS, TrypL/E treated (Gibco TrypLE Express Enzyme (1×), phenol red 12605028, Thermo Fisher Scientific), singularized, washed again with 1× PBS and filtered. All samples were resuspended in 100 μl Annexin V Binding buffer each, and stained in FACS tubes with Propidium iodide (PI, 1 mg/ml, P4864 Sigma‐Aldrich) and Annexin V‐FITC (50 μg/ml, 556,420, BD Biosciences). After an incubation for 10–15 min at RT (protected from light), 200 μl of cold Annexin V Binding buffer was added and samples were measured on a flow cytometer (LSRFortessa flow cytometer, BD Biosciences). Data analysis was performed with FlowJo v10.06 (BD Biosciences) and GraphPad Prism 8.0.1.

### NAC experiments

Cells were seeded in 96‐well plates (CytoOne) at a density of 2.5–5 × 10^4^ per ml in the respective cell culture media (RPMI with 10% FBS for adherent cells, Dulbecco's Modified Eagle's Medium with 10% FBS for LN229). After 24 h, cells were exposed to DHA and/or 5‐ALA with or without 1 h preincubation with freshly prepared NAC (dissolved in cell culture media, R10; Sigma Aldrich A2750‐10G). The ATP‐based CellTiter‐Glo Luminescent Cell Viability Assay (Promega, G7570) was used to measure viability according to manufacturer's instructions and luminescence was measured with the Tecan infinite 200Pro plate reader (Zurich, Switzerland). GraphPad Prism software (version 8.0.1) was used to determine the dose response curves and calculate the activity expressed as IC_50_ values. Results are given as means ± standard deviation (SD) and were normalized to untreated control cells. For clonogenic assays, cells were seeded in 24‐well plates (CytoOne) at a density of 1–5 × 10^3^ per well in 500 μl of the respective cell culture media (RPMI with 10% FBS for adherent cells, Dulbecco's Modified Eagle's Medium with 10% FBS for LN229). After 24 h, cells were exposed to the indicated drugs (DHA, 5‐ALA) with or without 1 h preincubation with freshly prepared NAC (dissolved in cell culture media, R10, Sigma Aldrich A2750‐10G). After 7 days cells were fixed with methanol, stained with crystal violet, and photographed with a macro lens on a Nikon D3200 camera (Minato). For quantification, crystal violet was eluted using 2% sodium dodecyl sulfate (SDS) and color absorbance was measured at 560 nm at the Tecan infinite 200Pro (Zurich, Switzerland). Values were analyzed using GraphPad Prism (version 8.0.1) and are given in arbitrary units (AU) as means ± SD normalized to the untreated control.

### Lipid peroxidation (BODIPY 581/591 C11 staining)

Cells were seeded at 2.5 × 10^5^ cells per ml of the respective cell culture media (RPMI with 10% FBS for adherent cells, Dulbecco's Modified Eagle's Medium with 10% FBS for LN229) in 6‐well plates (CytoOne). The next day, cells were exposed to DHA and/ or 5‐ALA at the indicated concentrations. Hydrogen peroxide (H_2_O_2_ solution, 30% [w/w] in H_2_O, H1009, Sigma‐Aldrich) was used as a positive control. After 24 h, 1 μM of the lipid peroxidation sensor BODIPY 581/591 C11 (Thermo Fisher Scientific, D3861) was added to the cells for 30 min. Afterward, three washing steps with 1x PBS were performed. Cells were then trypsinized, resuspended in cell culture media without FBS, and subjected to flow cytometry using b610/20 (Red) and b530/30 (FITC) channels. Autofluorescence of cells was subtracted from measured values, and fluorescence shifts from red (590 nm) to green (510 nm) were assessed as an indication of oxidation. Data analysis was performed with FlowJo v10.06 and GraphPad Prism 8.0.1.

### Animal experiments

All mice were bred, maintained, examined, and euthanized in accordance with institutional animal care guidelines and ethical animal license protocols approved by the legal authorities. Experimental animals were purchased from Charles River and housed at the Institute of Molecular Biotechnology (IMBA, Vienna, Austria), in a 12‐h light/dark cycle, with food and water *ad libitum*. Only female *Crl:NU*(*NCr*)*‐Foxn1nu* (Nude) mice group housed in individually ventilated cages were used for all experiments described. Animals were randomly assigned to experimental groups and checked daily by veterinary staff. Exclusion criteria for all assays were specified *a priori*; however, no animal was excluded. Experiments were performed in accordance with the ARRIVE guidelines. All animal experiments were approved by the Austrian Federal Ministry of Education, Science and Research (BMWFW‐66.015/0021‐WF/3b/2014) and conducted according to project license GZ 2020–0.762.422 and their amendments.

### Patient material

Patients with malignant brain tumors and/or their legal representatives treated at the Medical University of Vienna gave preoperative informed consent to participate in the study in all cases. The study was approved by the local institutional review board (IRB) of the Medical University of Vienna (EK Nr. 1244/2016, EK Nr: 1616/2020) according to the guidelines of the Helsinki Declaration developed by the World Medical Association and the Department of Health and Human Services Belmont Report.

### Glioblastoma PDX models

VBT529 or VBT531 glioblastoma cells were transduced with a Luc2‐iRFP lentiviral vector. Following FACS sorting, 1.5–2 × 10^5^ LUC2 expressing cells were injected into the brain or flank and brain of *Crl:NU*(*NCr*)*‐Foxn1nu* (Nude) mice. For orthotopic PDX, mice were anesthetized and restrained in a stereotaxic frame. The body temperature was monitored with a rectal thermometer and kept constant at 36°C by a heating pad. The skull was exposed, cleaned, and a small hole was drilled. Cells were injected at coordinates: AP‐1, ML‐2, DV‐3. To prevent backflow, the needle was left at the injection site for 6 min after the injection was finished. Mice were left to recover for at least 1 week after the surgery and during that time their drinking water was supplied with Baytril (Bayer) and Rimadyl (Pfizer). Tumor growth was monitored using the IVIS imaging system. Artesunate (ARS, A3731‐100MG, 88495‐63‐0 300 mg, Sigma) and 5‐ALA (5‐aminolevulinic acid hydrochloride, HY‐N0305, 5451‐09‐2 MedchemExpress) were administered intraperitoneally (IP) at the concentrations and timepoints indicated in the figure legends. 5‐ALA was dissolved in water, and ARS was dissolved in 5% NaHCO_3_ (144‐55‐8, 8551.1, Roth) in 0.9% NaCl (7647‐14‐5, 3570130, B. Braun). Compounds were not mixed prior to injection, and 5‐ALA and ARS were injected right after each other. For the comparisons between artesunate, 5‐ALA and artesunate + 5‐ALA, mice were 11 weeks old at the start of the experiment (surgery). For the comparison between artesunate and artesunate + 5‐ALA, mice were 24 weeks old at the start of the experiment (surgery).

### Metabolomics

Mice were treated with 120 mg/kg 5‐ALA via intraperitoneal injection 1 h prior to sacrifice. Brain and tumor samples were snap‐frozen in liquid nitrogen and homogenized in ice cold methanol:acetonitrile:water (2:2:1, v/v/v). Metabolite extracts were analyzed by reversed phase chromatography directly coupled to mass spectrometry (LC–MS/MS). For each sample, 1 μl was injected onto a Kinetex (Phenomenex) C18 column (100 Å, 150 × 2.1 mm) connected with a respective guard column, employing a 3‐min‐long linear gradient from 3% A (1% acetonitrile, 0.1% formic acid in water) to 95% B (0.1% formic acid in acetonitrile) at a flow rate of 80 μl/min, followed by isocratic elution for 25 min. Detection and quantification were done by selected reaction monitoring (SRM), employing a TSQ Altis mass spectrometer (Thermo Fisher Scientific) and using the following transitions in the positive ion mode: 210–122 *m*/*z* (porphobilinogen), 211–163 *m*/*z*, 563–504 *m*/*z* (protoporphyrin IX). Data interpretation was performed using Xcalibur (Thermo Fisher Scientific). Authentic standards were used for determining the optimal collision energies and for the confirmation of experimental retention times via standards added to the control sample.

### Statistics and reproducibility

All values are given as means ± SD, unless stated otherwise. Box and whisker plots depict the median and ranges from the first to the third quartile. All experiments were reproduced at two to seven independent times, with similar results. GraphPad Prism was used to generate figures and perform statistical analyses (GraphPad Software). An *a priori* sample size estimation was not performed. The experiments were not randomized. The investigators were not blinded to allocation during experiments and outcome assessment. Data were analyzed by using the unpaired two‐tailed Student's *t*‐test, as indicated. *P* < 0.05 was accepted as statistically significant.

**The paper explained**

**Problem**

The natural compound Artemisinin is some of the most widely used drugs against Malaria worldwide. Artemisinins are so‐called endoperoxides that damage proteins in cells and can thus act as toxins. These cytotoxic properties have not only been recognized to treat malaria but also for their anticancer properties. However, the exact mechanism of action of Artemisinins has only been poorly characterized.

**Results**

We used genome‐wide screens in yeast and haploid ESCs and found that a single pathway, namely mitochondrial heme production (also known as porphyrin biosynthesis), is accountable for Artemisinin's cytotoxicity in eukaryotic cells. We find that modulation of heme production is sufficient to increase or decrease Artemisinin's cytotoxicity in different cell types, including human cancer cells. We further tested the small molecule 5‐ALA, an enhancer of heme production, that is used in the clinic specifically to mark brain tumor cells during surgery, as it is metabolized specifically in cancer cells but not in surrounding tissue. We used a variety of different *in vitro* and *in vivo* in mouse and human model systems of brain cancer development and showed that 5‐ALA specifically sensitizes brain tumor cells to Artemisinins' cytotoxicity, without affecting normal tissue growth. We thus find that heme biosynthesis is the most critical molecular pathway for Artemisinin cytotoxicity in eukaryotic cells and could potentially be used for antibrain cancer therapy in combination with the heme enhancer 5‐ALA.

**Impact**

We combined an unbiased screening approach and several clinically relevant brain cancer model systems to suggest a novel translational sensitization strategy for human glioblastoma. We envisage to use a clinically approved small molecule together with one of the most widely used antimalarial compounds, Artemisinin, to treat highly malignant brain tumors, such as human glioblastomas, in human patient therapy.

## Author contributions

**Jasmin Taubenschmid‐Stowers:** Conceptualization; formal analysis; investigation; visualization; methodology; writing—original draft; writing—review and editing. **Michael Orthofer:** Formal analysis; investigation; visualization; methodology; writing—review and editing. **Anna Laemmerer:** Formal analysis; investigation; visualization; methodology; writing—review and editing. **Christian Krauditsch:** Investigation; methodology; writing—review and editing. **Marianna Rózsová:** Investigation. **Christian Studer:** Investigation. **Daniela Lötsch:** Formal analysis; investigation; visualization; methodology; writing—review and editing. **Johannes Gojo:** Investigation; methodology; writing—review and editing. **Lisa Gabler:** Investigation. **Matheus Dyczynski:** Investigation. **Thomas Efferth:** Investigation. **Astrid Hagelkruys:** Project administration. **Georg Widhalm:** Resources; funding acquisition; investigation. **Andreas Peyrl:** Resources; investigation. **Sabine Spiegl‐Kreinecker:** Resources. **Dominic Hoepfner:** Formal analysis; investigation; visualization; methodology; writing—review and editing. **Shan Bian:** Resources; methodology. **Moritz Horn:** Resources; investigation; project administration; writing—review and editing. **Walter Berger:** Resources; supervision; funding acquisition; investigation; methodology; writing—review and editing. **Juergen A Knoblich:** Resources; funding acquisition; methodology. **Ulrich Elling:** Conceptualization; formal analysis; methodology. **Josef M Penninger:** Conceptualization; resources; supervision; funding acquisition; writing—review and editing.

## Disclosure and competing interests statement

JMP is founder shareholder and supervisory board member of JLP Health. UE and MH are shareholders of JLP Health. MO, MR, and MD are employees of JLP Health. JLP Health develops a combinatorial cancer therapy based on 5‐ALA and artesunate.

## Supporting information



Appendix S1

Expanded View Figures PDF

PDF+

Dataset EV1

Dataset EV2

## Data Availability

This study includes no data deposited in external repositories.
