# Supplementary material for: A whole‐genome scan for Artemisinin cytotoxicity reveals a novel therapy for human brain tumors
Source: EMBO Mol Med. 2023 Feb 6;15(3):e16959. doi: 10.15252/emmm.202216959 (PMC10237280; doi:10.15252/emmm.202216959)
Supplement: Supplementary file 1 — Appendix S1 [file EMMM-15-e16959-s003.pdf]

Appendix Table S1. Exact P values

Figure 3E

Multiple unpaired t tests	Below threshold?	Summary	Adjusted P Value
no DHA vs DHA (-)	No	ns	0.986176
no DHA vs DHA + ALA	Yes	***	0.000593
no DHA vs DHA + Ppox	No	ns	>0.99999999
no DHA vs DHA + ALA + Ppox	No	ns	0.194485

Figure 3F

Multiple unpaired t tests	Below threshold?	Summary	Adjusted P Value
no DHA vs DHA (-)	No	ns	>0.99999999
no DHA vs DHA + ALA	Yes	**	0.007646
no DHA vs DHA + Ppox	No	ns	>0.99999999
no DHA vs DHA + ALA + Ppox	No	ns	>0.99999999

Figure 4E

Multiple unpaired t-tests	Below threshold?	Summary	Adjusted P Value
no ALA vs 0.0625mM 5-ALA + 0.0 DHA	No	ns	0.371998
no ALA vs 0.0625mM 5-ALA + 0.125 DHA	No	ns	0.334582
no ALA vs 0.0625mM 5-ALA + 0.5 DHA	Yes	*	0.027738
no ALA vs 0.0625mM 5-ALA + 1.0 DHA	No	ns	0.051646

Figure 4F

Multiple unpaired t-tests	Below threshold?	Summary	Adjusted P Value
no 5-ALA vs 0.0625mM 5-ALA + 0.0 DHA	No	ns	0.959208
no 5-ALA vs 0.0625mM 5-ALA + 0.125 DHA	No	ns	0.966479
no 5-ALA vs 0.0625mM 5-ALA + 0.5 DHA	No	ns	0.283520
no 5-ALA vs 0.0625mM 5-ALA + 1.0 DHA	Yes	*	0.043791

Figure 5C

Multiple unpaired t-tests	Below threshold?		Adjusted P Value
no 5-ALA	No	ns	0.650821
0.125 DHA	No	ns	0.134240
0.5 DHA	Yes	**	0.006084
1.0 DHA	Yes	**	0.001684

Figure 6C

Multiple unpaired t-tests	Discovery?	Summary	Adjusted P Value
control vs. 0.5mM 5-ALA + 0.0 DHA	No	ns	0.064467
control vs. 0.5mM 5-ALA + 0.5 DHA	Yes	***	<0.000001
control vs. 0.5mM 5-ALA + 1.0 DHA	Yes	***	<0.000001
control vs. 0.5mM 5-ALA + 5.0 DHA	Yes	***	<0.000001
control vs. 1mM 5-ALA + 0 DHA	No	ns	0.728198
control vs. 1mM 5-ALA + 1 DHA	Yes	***	<0.000001
control vs. 1mM 5-ALA + 5 DHA	Yes	***	<0.000001
control vs. 1mM 5-ALA + 10 DHA	Yes	***	<0.000001

Figure 6D

Bonferroni's multiple comparisons test	Below threshold?	Summary	Adjusted P Value
control vs. DMSO	No	ns	>0.99
control vs. 25 µM DHA	Yes	***	<0.001
control vs. 5 mM 5-Ala	Yes	**	0.002000
control vs. DHA+5-Ala	Yes	***	<0.001
control vs. 100 µM H2O2	No	ns	>0.99
25 µM DHA vs. DHA+5-Ala	Yes	***	<0.001

5 mM 5-Ala vs. DHA+5-Ala	Yes	***	<0.001

Figure 7B

two-way ANOVA followed by Bonferroni's multiple comparisons test	Below threshold?	Summary	Adjusted P Value
Day 31: Control vs. 5-ALA	No	ns	0.527600
Day 31: Control vs. ARS	No	ns	0.121400
Day 31: Control vs. 5-ALA + ARS	Yes	****	<0.0001

Figure 7C

Student's one-tailed, unpaired t-test	Below threshold?	Summary	Adjusted P Value
Day 7: Control vs. 5-ALA + ARS	No	ns	0.472931
Day 11: Control vs. 5-ALA + ARS	No	ns	0.228438
Day 18: Control vs. 5-ALA + ARS	No	ns	0.061445
Day 24: Control vs. 5-ALA + ARS	Yes	*	0.039790
Day 32: Control vs. 5-ALA + ARS	Yes	**	0.007276
Day 39: Control vs. 5-ALA + ARS	Yes	*	0.015541
Day 46: Control vs. 5-ALA + ARS	Yes	**	0.003186

Figure 7E

Student's one-tailed, unpaired t-test	Below threshold?	Summary	Adjusted P Value
Day 7: Control vs. 5-ALA + ARS	No	ns	0.405833
Day 11: Control vs. 5-ALA + ARS	Yes	**	0.006570
Day 18: Control vs. 5-ALA + ARS	Yes	***	0.000047
Day 24: Control vs. 5-ALA + ARS	Yes	**	0.002020
Day 32: Control vs. 5-ALA + ARS	Yes	*	0.012704
Day 39: Control vs. 5-ALA + ARS	Yes	*	0.019824
Day 46: Control vs. 5-ALA + ARS	Yes	*	0.027759

Figure 7G

Log-rank (Mantel-Cox) test	Below threshold?	Summary	Adjusted P Value
Control vs. 5-ALA + ARS	Yes	***	0.000500

Figure 7H left

Student's one-tailed, unpaired t-test	Below threshold?	Summary	Adjusted P Value
PBG levels (5ALA treated): Brain vs. Tumor	Yes	*	0.028000

Figure 7H right

Student's one-tailed, unpaired t-test	Below threshold?	Summary	Adjusted P Value
PPIX levels (5ALA treated): Brain vs. Tumor	Yes	*	0.019000

Figure EV2 G

Multiple unpaired t tests	Below threshold?	Summary	Adjusted P Value
no DHA vs DHA (-)	No	ns	0.200233
no DHA vs DHA + ALA	Yes	*	0.021779
no DHA vs DHA + Ppox	No	ns	0.999381
no DHA vs DHA + ALA + Ppox	No	ns	0.200233

Figure EV2 H

Multiple unpaired t tests	Below threshold?	Summary	Adjusted P Value
no DHA vs DHA (-)	No	ns	0.996965
no DHA vs DHA + ALA	Yes	*	0.017157
no DHA vs DHA + Ppox	No	ns	0.996965
no DHA vs DHA + ALA + Ppox	No	ns	0.996965

Figure EV2 I

Multiple unpaired t tests	Below threshold?	Summary	Adjusted P Value

no DHA vs DHA (-)	No	ns	0.911831
no DHA vs DHA + ALA	Yes	*	0.036731
no DHA vs DHA + Ppox	No	ns	0.905315
no DHA vs DHA + ALA + Ppox	No	ns	0.905315

Figure EV2 L

multiple unpaired t tests	Below threshold?	Summary	Adjusted P Value
no ALA vs 0.0625mM 5-ALA + 0.0 DHA	No	ns	0.854506
no ALA vs 0.0625mM 5-ALA + 0.125 DHA	No	ns	0.500587
no ALA vs 0.0625mM 5-ALA + 0.5 DHA	Yes	**	0.002698
no ALA vs 0.0625mM 5-ALA + 1.0 DHA	No	ns	0.627216

Figure EV3 G

multiple unpaired t tests	Below threshold?	Summary	Adjusted P Value
no ART no ALA vs. 0.5 ART no ALA Casp3+ GFP+ tumour cells	No	ns	0.273827
no ART no ALA vs.1.0 ART no ALA Casp3+ GFP+ tumour cells	Yes	**	0.012248
no ART 0.0625 ALA vs. 0.5 ART 0.0625 ALA Casp3+ GFP+ tumour cells	No	ns	0.143251
no ART 0.0625 ALA vs. 1.0 ART 0.0625 ALA Casp3+ GFP+ tumour cells	Yes	***	0.000451

Figure EV3 H

multiple unpaired t tests	Below threshold?	Summary	Adjusted P Value
no ART no ALA vs. 0.5 ART no ALA Ki67+ GFP+ tumour cells	No	ns	>0.99999999
no ART no ALA vs.1.0 ART no ALA Ki67+ GFP+ tumour cells	No	ns	0.552919
no ART 0.0625 ALA vs. 0.5 ART 0.0625 ALA Ki67+ GFP+ tumour cells	No	ns	>0.99999999
no ART 0.0625 ALA vs. 1.0 ART 0.0625 ALA Ki67+ GFP+ tumour cells	No	ns	0.369626

Figure EV4 B

multiple unpaired t tests	Below threshold?	Summary	Adjusted P Value
no 5-ALA	No	ns	0.792109
0.125 DHA	No	ns	0.413177
0.5 DHA	No	ns	0.792109
1.0 DHA	Yes	*	0.019061

Figure EV4 F

Multiple unpaired t-tests	Discovery?	Summary	Adjusted P Value
control vs. 0.1mM 5-ALA + 0.0 DHA	No	ns	0.164376
control vs. 0.1mM 5-ALA + 0.5 DHA	No	ns	0.114962
control vs. 0.1mM 5-ALA + 1.0 DHA	Yes	**	0.004831
control vs. 0.1mM 5-ALA + 5.0 DHA	No	ns	0.164376
control vs. 0.5mM 5-ALA + 0.0 DHA	No	ns	0.158229
control vs. 0.5mM 5-ALA + 0.5 DHA	No	ns	0.119754
control vs. 0.5mM 5-ALA + 1.0 DHA	Yes	***	0.000056
control vs. 0.5mM 5-ALA + 5.0 DHA	Yes	***	0.000020

Figure EV4 G

Multiple unpaired t-tests	Discovery?	Summary	Adjusted P Value
control vs. 0.1mM 5-ALA + 0.0 DHA	No	ns	0.131099
control vs. 0.1mM 5-ALA + 0.5 DHA	No	ns	>0.99999999

control vs. 0.1mM 5-ALA + 1.0 DHA	No	ns	>0.99999999
control vs. 0.1mM 5-ALA + 5.0 DHA	No	ns	0.050978
control vs. 0.5mM 5-ALA + 0.0 DHA	Yes	***	0.000013
control vs. 0.5mM 5-ALA + 0.5 DHA	No	ns	>0.99999999
control vs. 0.5mM 5-ALA + 1.0 DHA	No	ns	>0.99999999
control vs. 0.5mM 5-ALA + 5.0 DHA	Yes	***	0.000010

Figure EV4 H

Multiple unpaired t-tests	Discovery?	Summary	Adjusted P Value
control vs. 0.1mM 5-ALA + 0 DHA	No	ns	0.850781
control vs. 0.1mM 5-ALA + 1 DHA	No	ns	0.169731
control vs. 0.1mM 5-ALA + 5 DHA	Yes	***	0.000317
control vs. 0.1mM 5-ALA + 10 DHA	Yes	***	0.000003
control vs. 0.5mM 5-ALA + 0 DHA	Yes	***	0.000022
control vs. 0.5mM 5-ALA + 1 DHA	Yes	***	0.000006
control vs. 0.5mM 5-ALA + 5 DHA	Yes	***	<0.000001
control vs. 0.5mM 5-ALA + 10 DHA	Yes	***	<0.000001

Figure EV4 J

Multiple unpaired t-tests	Discovery?	Summary	Adjusted P Value
control vs. 0.5mM 5-ALA + 0 DHA	No	ns	0.144486
control vs. 0.5mM 5-ALA + 1 DHA	Yes	***	0.000943
control vs. 0.5mM 5-ALA + 5 DHA	No	ns	0.144486
control vs. 0.5mM 5-ALA + 10 DHA	Yes	***	<0.000001
control vs. 1mM 5-ALA + 0 DHA	No	ns	0.375535
control vs. 1mM 5-ALA + 1 DHA	Yes	***	0.000278
control vs. 1mM 5-ALA + 5 DHA	No	ns	0.180525
control vs. 1mM 5-ALA + 10 DHA	Yes	***	<0.000001

Figure EV5 A

Bonferroni's multiple comparisons test	Below threshold?	Summary	Adjusted P Value
control vs. DMSO	No	ns	0.290000
control vs. 25 μM DHA	Yes	***	<0.001
control vs. 1 mM 5-Ala	No	ns	>0.99
control vs. DHA+5-Ala	Yes	***	<0.001
25 μM DHA vs. DHA+5-Ala	Yes	***	<0.001
1 mM 5-Ala vs. DHA+5-Ala	Yes	***	<0.001

Figure EV5 B

Bonferroni's multiple comparisons test	Below threshold?	Summary	Adjusted P Value
control vs. DMSO	Yes	***	<0.001
control vs. 25 μM DHA	No	ns	>0.99
control vs. 1 mM 5-Ala	Yes	***	<0.001
control vs. DHA+5-Ala	Yes	***	<0.001
25 μM DHA vs. 1 mM 5-Ala	Yes	***	<0.001
25 μM DHA vs. DHA+5-Ala	Yes	***	<0.001
1 mM 5-Ala vs. DHA+5-Ala	Yes	***	<0.001

Figure EV5 C

Bonferroni's multiple comparisons test	Below threshold?	Summary	Adjusted P Value
control vs. DMSO	No	ns	>0.99
control vs. 25 μM DHA	Yes	***	<0.001
control vs. 5 mM 5-Ala	Yes	**	0.002000
control vs. DHA+5-Ala	Yes	***	<0.001
control vs. 100 μM H2O2	No	ns	>0.99
25 μM DHA vs. 5 mM 5-Ala	No	ns	0.560000

25 µM DHA vs. DHA+5-Ala	Yes	***	<0.001
5 mM 5-Ala vs. DHA+5-Ala	Yes	***	<0.001

Figure EV5 D

Bonferroni's multiple comparisons test	Below threshold?	Summary	Adjusted P Value
control vs. DMSO	No	ns	>0.99
control vs. 25 µM DHA	Yes	***	<0.001
control vs. 5 mM 5-Ala	Yes	**	0.006000
control vs. DHA+5-Ala	Yes	***	<0.001
control vs. 500 µM H2O2	Yes	***	<0.001
25 µM DHA vs. DHA+5-Ala	Yes	***	<0.001
5 mM 5-Ala vs. DHA+5-Ala	Yes	***	<0.001

Figure EV5 J

two-way ANOVA followed by Bonferroni's multiple comparisons test	Below threshold?	Summary	Adjusted P Value
Day 13: control vs. 5 ALA	No	ns	>0.99999999
Day 13: control vs. ARS	No	ns	>0.99999999
Day 13: control vs. 5ALA + ARS	No	ns	>0.99999999
Day 14: control vs. 5 ALA	No	ns	>0.99999999
Day 14: control vs. ARS	No	ns	>0.99999999
Day 14: control vs. 5ALA + ARS	No	ns	>0.99999999
Day 15: control vs. 5 ALA	No	ns	>0.99999999
Day 15: control vs. ARS	No	ns	>0.99999999
Day 15: control vs. 5ALA + ARS	No	ns	>0.99999999
Day 16: control vs. 5 ALA	No	ns	>0.99999999
Day 16: control vs. ARS	No	ns	>0.99999999
Day 16: control vs. 5ALA + ARS	No	ns	>0.99999999
Day 17: control vs. 5 ALA	No	ns	>0.99999999
Day 17: control vs. ARS	No	ns	>0.99999999
Day 17: control vs. 5ALA + ARS	No	ns	>0.99999999
Day 20: control vs. 5 ALA	No	ns	>0.99999999
Day 20: control vs. ARS	No	ns	>0.99999999
Day 20: control vs. 5ALA + ARS	No	ns	0.925400
Day 21: control vs. 5 ALA	No	ns	>0.99999999
Day 21: control vs. ARS	No	ns	>0.99999999
Day 21: control vs. 5ALA + ARS	No	ns	>0.99999999
Day 22: control vs. 5 ALA	No	ns	>0.99999999
Day 22: control vs. ARS	No	ns	0.908200
Day 22: control vs. 5ALA + ARS	No	ns	>0.99999999
Day 23: control vs. 5 ALA	No	ns	>0.99999999
Day 23: control vs. ARS	No	ns	0.937000
Day 23: control vs. 5ALA + ARS	No	ns	>0.99999999
Day 24: control vs. 5 ALA	No	ns	0.929500
Day 24: control vs. ARS	No	ns	>0.99999999
Day 24: control vs. 5ALA + ARS	No	ns	>0.99999999
Day 27: control vs. 5 ALA	No	ns	0.381900
Day 27: control vs. ARS	No	ns	0.404100
Day 27: control vs. 5ALA + ARS	No	ns	0.117000
Day 28: control vs. 5 ALA	No	ns	0.611700
Day 28: control vs. ARS	No	ns	0.564400
Day 28: control vs. 5ALA + ARS	No	ns	0.277400
Day 29: control vs. 5 ALA	No	ns	0.254400
Day 29: control vs. ARS	No	ns	0.357300
Day 29: control vs. 5ALA + ARS	No	ns	0.311200
Day 30: control vs. 5 ALA	No	ns	0.486400

Day 30: control vs. ARS	No	ns	0.312100
Day 30: control vs. 5ALA + ARS	No	ns	0.385600
Day 31: control vs. 5 ALA	No	ns	0.411800
Day 31: control vs. ARS	No	ns	0.722200
Day 31: control vs. 5ALA + ARS	No	ns	>0.99999999

Figure EV5 K

two-way ANOVA followed by Bonferroni's multiple comparisons test	Below threshold?	Summary	Adjusted P Value
Day 7: control vs. 5ALA + ARS	No	ns	>0.99999999
Day 8: control vs. 5ALA + ARS	No	ns	>0.99999999
Day 9: control vs. 5ALA + ARS	No	ns	>0.99999999
Day 10: control vs. 5ALA + ARS	No	ns	>0.99999999
Day 11: control vs. 5ALA + ARS	No	ns	>0.99999999
Day 14: control vs. 5ALA + ARS	No	ns	>0.99999999
Day 15: control vs. 5ALA + ARS	No	ns	>0.99999999
Day 16: control vs. 5ALA + ARS	No	ns	>0.99999999
Day 18: control vs. 5ALA + ARS	No	ns	>0.99999999
Day 21: control vs. 5ALA + ARS	No	ns	>0.99999999
Day 22: control vs. 5ALA + ARS	No	ns	>0.99999999
Day 23: control vs. 5ALA + ARS	No	ns	>0.99999999
Day 24: control vs. 5ALA + ARS	No	ns	>0.99999999
Day 28: control vs. 5ALA + ARS	No	ns	>0.99999999
Day 29: control vs. 5ALA + ARS	No	ns	>0.99999999
Day 30: control vs. 5ALA + ARS	No	ns	>0.99999999
Day 31: control vs. 5ALA + ARS	No	ns	>0.99999999
Day 33: control vs. 5ALA + ARS	No	ns	>0.99999999
Day 35: control vs. 5ALA + ARS	No	ns	>0.99999999
Day 36: control vs. 5ALA + ARS	No	ns	>0.99999999
Day 37: control vs. 5ALA + ARS	No	ns	>0.99999999
Day 38: control vs. 5ALA + ARS	No	ns	>0.99999999

Figure EV5 L

Student's one-tailed, unpaired t-test	Below threshold?	Summary	Adjusted P Value
Day 6: Control vs. 5-ALA + ARS	No	ns	0.403257
Day 11: Control vs. 5-ALA + ARS	Yes	*	0.049907
Day 18: Control vs. 5-ALA + ARS	No	ns	0.053896
Day 25: Control vs. 5-ALA + ARS	Yes	*	0.027773
Day 31: Control vs. 5-ALA + ARS	Yes	*	0.023992
Day 39: Control vs. 5-ALA + ARS	Yes	*	0.038073
Day 46: Control vs. 5-ALA + ARS	Yes	*	0.039888
Day 53: Control vs. 5-ALA + ARS	Yes	*	0.029304
Day 60: Control vs. 5-ALA + ARS	Yes	*	0.034771
Day 66: Control vs. 5-ALA + ARS	No	ns	0.076268

Figure EV5 N

Student's one-tailed, unpaired t-test	Below threshold?	Summary	Adjusted P Value
Day 6: Control vs. 5-ALA + ARS	No	ns	0.417689
Day 11: Control vs. 5-ALA + ARS	No	ns	0.093611
Day 18: Control vs. 5-ALA + ARS	Yes	**	0.002190
Day 25: Control vs. 5-ALA + ARS	Yes	***	0.000445
Day 31: Control vs. 5-ALA + ARS	Yes	**	0.001004
Day 39: Control vs. 5-ALA + ARS	Yes	**	0.002604
Day 46: Control vs. 5-ALA + ARS	Yes	**	0.004013
Day 53: Control vs. 5-ALA + ARS	Yes	**	0.001531
Day 60: Control vs. 5-ALA + ARS	Yes	**	0.001674

Day 66: Control vs. 5-ALA + ARS	Yes	**	0.001322

Figure EV5 P

Log-rank (Mantel-Cox) test	Below threshold?	Summary	Adjusted P Value
Control vs. 5-ALA + ARS	Yes	***	0.000500

Figure EV5 Q

two-way ANOVA followed by Bonferroni's multiple comparisons test	Below threshold?	Summary	Adjusted P Value
Day 7: Control vs. 5-ALA + ARS	No	ns	>0.99999999
Day 8: Control vs. 5-ALA + ARS	No	ns	>0.99999999
Day 9: Control vs. 5-ALA + ARS	No	ns	>0.99999999
Day 10: Control vs. 5-ALA + ARS	No	ns	>0.99999999
Day 14: Control vs. 5-ALA + ARS	No	ns	>0.99999999
Day 15: Control vs. 5-ALA + ARS	No	ns	>0.99999999
Day 16: Control vs. 5-ALA + ARS	No	ns	>0.99999999
Day 17: Control vs. 5-ALA + ARS	No	ns	>0.99999999
Day 18: Control vs. 5-ALA + ARS	No	ns	>0.99999999
Day 21: Control vs. 5-ALA + ARS	No	ns	>0.99999999
Day 22: Control vs. 5-ALA + ARS	No	ns	>0.99999999
Day 23: Control vs. 5-ALA + ARS	No	ns	>0.99999999
Day 25: Control vs. 5-ALA + ARS	No	ns	>0.99999999
Day 28: Control vs. 5-ALA + ARS	No	ns	>0.99999999
Day 29: Control vs. 5-ALA + ARS	No	ns	>0.99999999
Day 30: Control vs. 5-ALA + ARS	No	ns	>0.99999999
Day 31: Control vs. 5-ALA + ARS	No	ns	>0.99999999
Day 35: Control vs. 5-ALA + ARS	No	ns	>0.99999999
Day 36: Control vs. 5-ALA + ARS	No	ns	>0.99999999
Day 37: Control vs. 5-ALA + ARS	No	ns	>0.99999999
Day 38: Control vs. 5-ALA + ARS	No	ns	0.713100
Day 40: Control vs. 5-ALA + ARS	No	ns	0.231500
Day 42: Control vs. 5-ALA + ARS	No	ns	0.500600
Day 43: Control vs. 5-ALA + ARS	No	ns	0.138200
Day 44: Control vs. 5-ALA + ARS	Yes	*	0.022200
Day 45: Control vs. 5-ALA + ARS	Yes	**	0.003500
Day 49: Control vs. 5-ALA + ARS	No	ns	0.443400
Day 50: Control vs. 5-ALA + ARS	No	ns	>0.99999999
Day 51: Control vs. 5-ALA + ARS	No	ns	>0.99999999
Day 52: Control vs. 5-ALA + ARS	No	ns	>0.99999999
Day 56: Control vs. 5-ALA + ARS	No	ns	>0.99999999
Day 57: Control vs. 5-ALA + ARS	No	ns	>0.99999999
Day 58: Control vs. 5-ALA + ARS	No	ns	>0.99999999
Day 59: Control vs. 5-ALA + ARS	No	ns	>0.99999999
Day 60: Control vs. 5-ALA + ARS	No	ns	>0.99999999

Figure EV5 R left

Student's one-tailed, unpaired t-test	Below threshold?	Summary	Adjusted P Value
PBG levels (5ALA treated): Brain vs. Tumor	Yes	**	0.006000

Figure EV5 R right

Student's one-tailed, unpaired t-test	Below threshold?	Summary	Adjusted P Value
PPIX levels (5ALA treated): Brain vs. Tumor	Yes	**	0.008000
