# Supplementary material for: A whole‐genome scan for Artemisinin cytotoxicity reveals a novel therapy for human brain tumors
Source: EMBO Mol Med. 2023 Feb 6;15(3):e16959. doi: 10.15252/emmm.202216959 (PMC10237280; doi:10.15252/emmm.202216959)
Supplement: Supplementary file 2 — Expanded View Figures PDF [file EMMM-15-e16959-s006.pdf]

Expanded View Figures

Figure EV1. Identification of essential host cell factors for dihydroartemisinin cytotoxicity in yeast and murine ESCs.

A IC_{50} curves of yeast cells grown in two different batches of Artemisinin. Batch one was freshly added from the DMSO stock solution before the start of the experiment (triangles), the other one was added 60 h before and stored at room temperature (stars). The survival curves and IC_{50} values of treated cells are indistinguishable.

B Minimal inhibitory concentration (MIC) of dihydroartemisinin (DHA) on solid medium. Approximately 1,000 haploid wild-type strain cells were plated per well at different concentrations of DHA and imaged after 72 h.

C HIP pool growth curves over 20 cell generations with three back dilutions. Whereas DMSO control cells divide at similar rates, cells with 1× or 1.5× IC_{30} concentrations of DHA show reduced proliferation after each back dilution. Absorption was measured at 595 nm.

D Colony formation assay of DHA-treated yeast cells on YPD and YPEG plates. Cells were pretreated with DMSO or 1,000 μM DHA on solid medium and re-streaked onto glucose (YPD, fermentable carbon source) or ethanol/glycerol containing medium (YPEG, nonfermentable carbon source).

E Mitochondrial (MitoTracker) staining of DMSO (upper panel) or DHA (1,000 μM, lower panel) pretreated cells (after 72 h recovery on YPD). Scale bar 5 μm.

F MIC determination of DHA in homozygous and heterozygous tim18 and tom70 mutant yeast clones. Approximately 1,000 cells were plated per well, treated for 72 h and imaged.

G, H Cell survival of DHA-treated (G) mouse ESCs and (H) primary mouse fibroblasts (MEF p3), as well as mouse (B16F10—skin melanoma, 4T1—breast cancer) and human (MDA-MB-231—triple negative epithelial breast cancer, Mcf7—estrogen-/progesterone receptor-positive/HER2-negative breast cancer, Panc1—pancreatic) cancer cells. Viability was assessed after 48 h of treatment using Alamar Blue staining.

I Porphyrin biosynthesis pathway component integrations sites from the DHA screens in haploid ESCs. Genomic locations and targeted introns, exons of porphyrin biosynthesis genes (i.e., enzymes, bold), as well as retroviral (upper panel) or Tol2 transposon (lower panel) integrations sites (vertical bars) are shown on the forward (black) and reverse (gray) strands. The chemical structures of the substrates and products are shown for each enzyme reaction.

© 2023 The Authors

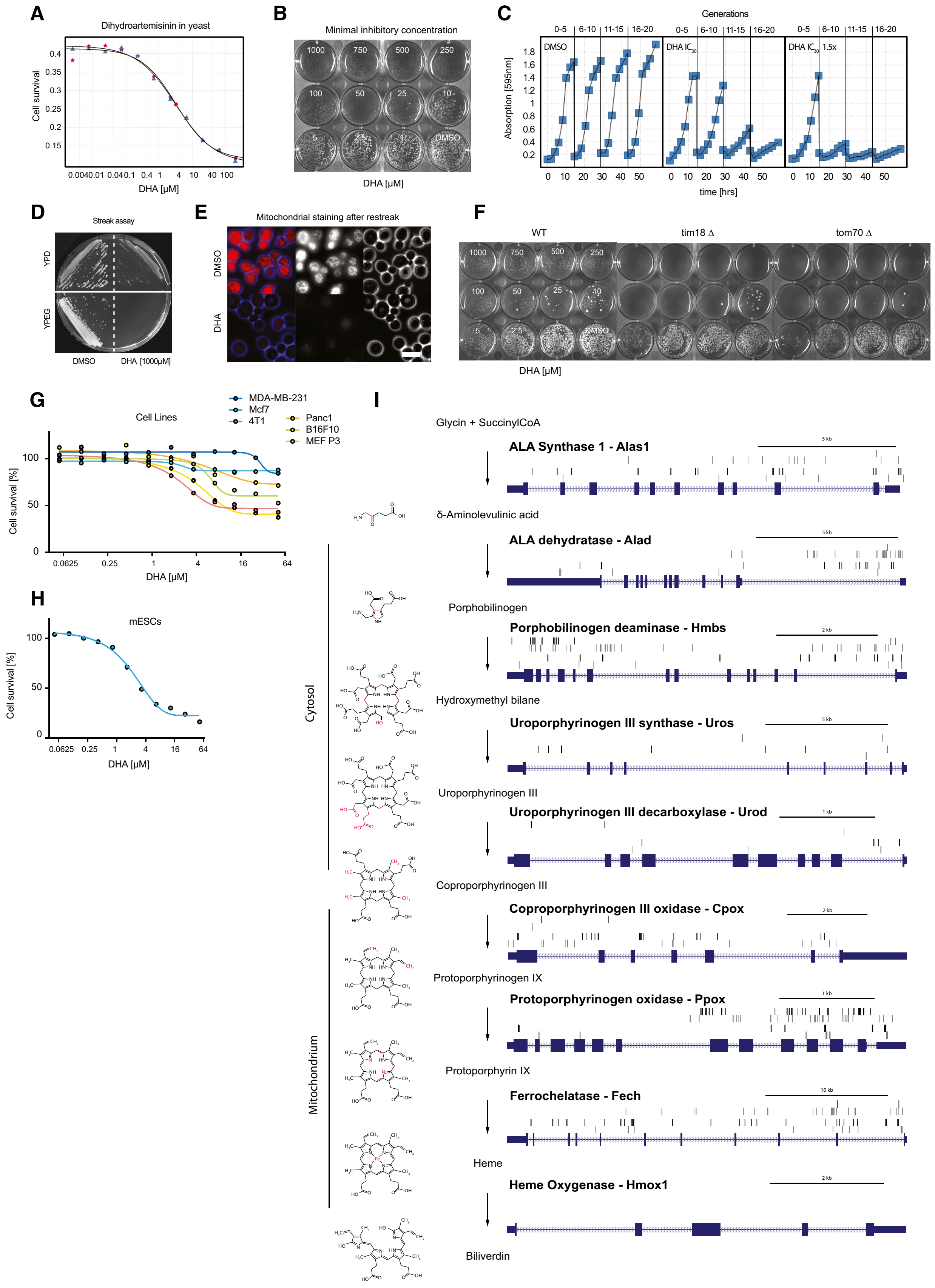

Figure EV1.

 © 2023 The Authors

Figure EV2. Modulation of porphyrin biosynthesis changes dihydroartemisinin cytotoxicity in cells and organoids.

A Cell viability of DHA-treated mouse cancer cells (4T1) in the presence and absence of 5-ALA (0.5 mM). Alamar Blue was used to determine cell survival after 72 h of treatment.

B Cell survival of DHA treated mouse (B16F10) and human (Mcf7) cancer cells, in the presence and absence of 5-ALA (0.5 mM). Alamar Blue was used to determine viability after 48 h.

C, D Cell survival of DHA treated primary human glioblastoma cells (BTL53, BTL1333) in the presence or absence of 5-ALA. CellTiter-Glo was used to assess viability, at 72 h. Experiment was performed in triplicate and repeated once. Values are mean ± SD.

E, F ROS/DHE staining and flow cytometry analysis of piperlongumine (PIP) or DHA (DHA) treated (E) Jurkat T cells and (F) HL-60 cells (48 h). Experiments were performed in triplicate. Values are mean ± SD.

G, H, I (G) ROS levels (DHE staining, PE 582/15 nm—MFI), (H) cell survival and (I) JC-1 levels in DHA (0.5 μM), 5-ALA (0.25 mM), or Ppox inhibitor (10 μM) treated Jurkat T-cells, 48 h. DHE fluorescence, relative cell numbers, and percentages of JC-1-negative cells were assessed using flow cytometry and automated high throughput cell counting. (G–I) All experiments were performed in triplicate and repeated once (JC-1) or twice (DHE, cell survival). Values are means ± SD.

J Cell survival of dissociated tumor organoids (central nervous system primitive neuroectodermal tumor (CNS-PNET-like) neoplasm model, c-MYC overexpression). Quantification of GFP^+ tumor cells and GFP-control cells of treated organoids normalized to control (DMSO) using flow cytometry are shown. Data are shown as box plots (25th–75th percentiles, median). The experiment was performed in triplicate and repeated twice.

K Representative fluorescence (left panel) and brightfield (right panel) images of control (DMSO), 5-ALA (0.0625 mM), DHA (1 μM), or 5-ALA + DHA (0.0625 mM and 1 μM) treated cerebral tumor organoids. Scale bar 500 μm.

L Quantification of GFP-positive tumor areas on day 5 of treatment, as compared to day 1. Box plots (25th–75th percentiles, median) of data are shown. The experiments were performed in triplicate each, repeated five times. Student's *t*-test was used to determine significance; $*P < 0.05$.

M Representative fluorescent (anti-GFP, DAPI) and H&E (hematoxylin and eosin stained) images of fixed cryo-sections of control and treated tumor organoids (CNS-PNET-like neoplasm). Regions of rosette-like structures (R) and tumor tissue (T) are indicated and magnified (6.8×). Top panels show GFP-labeled tumor cells (green) and DAPI counterstaining (blue) to image nuclei. Scale bar 500 μm.

© 2023 The Authors

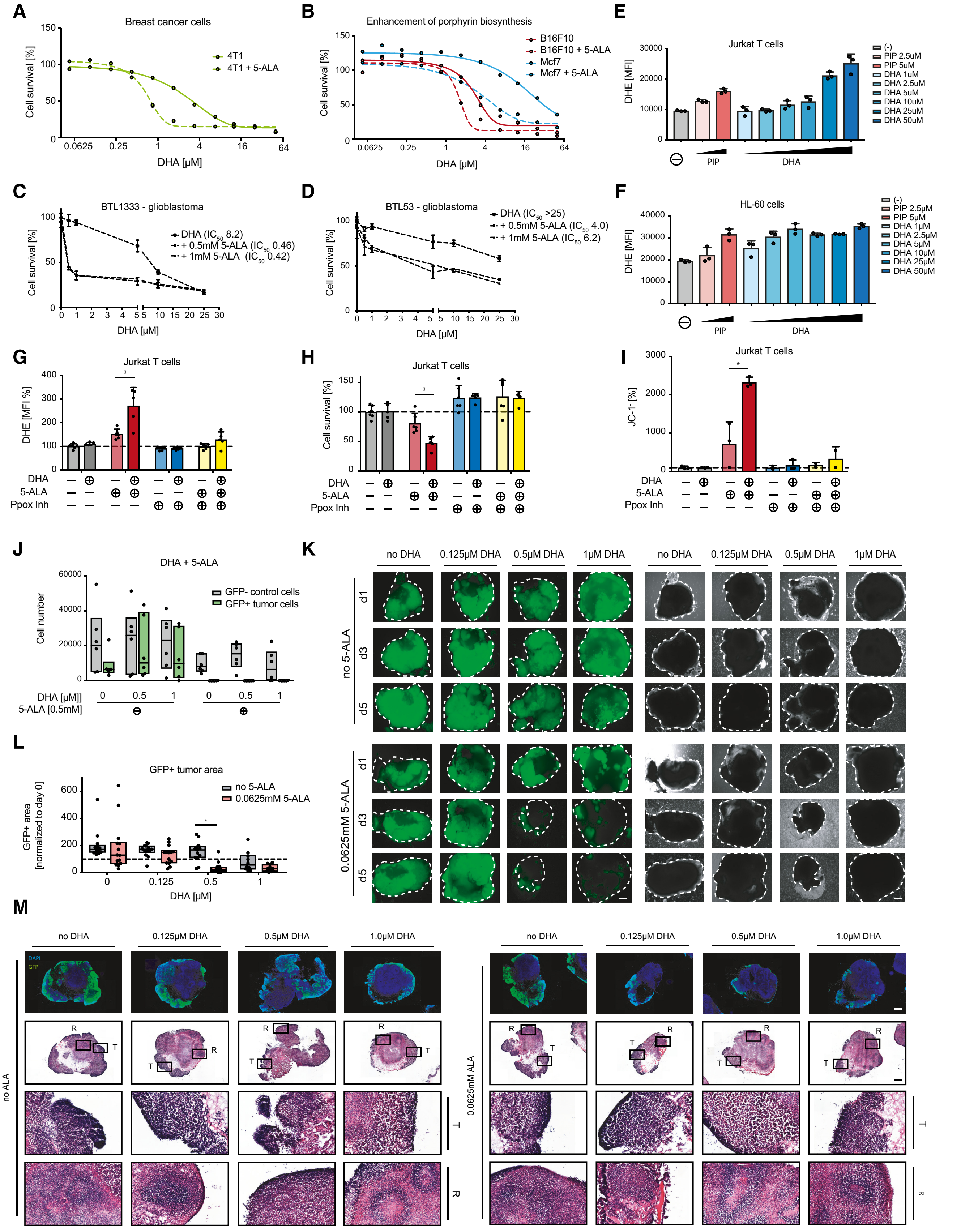

Figure EV2.

© 2023 The Authors

Figure EV3. Dihydroartemisinin and 5-ALA treatment of GFP+ tumor organoids induces ROS, DNA damage, and cell death.

A, B Representative images of DAPI (blue), anti-GFP (green), and (A) anti-MAP2 (red) or (B) anti-SOX2 (magenta) stained sections of control and DHA + 5-ALA treated cerebral tumor organoids (central nervous system primitive neuroectodermal tumor (CNS-PNET-like) neoplasm model, c-MYC overexpression). Scale bar 500 μm.

C Representative images of anti-Sox2 (magenta), anti-GFP (green), and DAPI (blue) stained sections of untreated and DHA and 5-ALA-treated organoids. Scale bar 50 μm.

D Representative FACS plots of ROS/DHE stained dissociated tumor organoids (CNS-PNET-like neoplasm, c-MYC overexpression). Flow cytometry analyses (PE, 582/15 nm) of GFP^+ tumor cells and GFP- wild-type cells are shown. The experiment was repeated twice.

E Representative images and analysis masks of γH2AX, GFP, and DAPI stained and scanned tumor organoid slides. Scale bar 500 μm.

F Representative microscopy images of γH2AX, GFP, and DAPI stained tumor organoid sections. Arrows indicate γH2AX-positive cells. Scale bar 50 μm.

G, H (G) Quantification of cleaved Caspase 3 (Casp3) and (H) Ki67-positive cells in 5-ALA- and DHA-treated tumor organoids (CNS-PNET-like neoplasm). Per condition and group, 3 organoids, 6 sections each, were stained with antibodies to detect cleaved Caspase 3 or Ki67. Sections were imaged using a high-magnification fluorescent scanner and 25 regions of interest (ROI, 2,500 μm^2) were chosen and analyzed. The numbers of Casp3- or Ki67-positive cells per GFP-positive or GFP-negative area were analyzed and are shown as box plots (median, 25th–75th percentiles).

I Representative images of anti-Casp3 (red), anti-GFP (green), and DAPI (blue) stained sections of brain tumor organoids. Scale bar 50 μm.

Data information: (G, H) Student's *t*-test was used to analyze significance; n.s., nonsignificant, $^{**}P < 0.01$, $^{***}P < 0.001$.

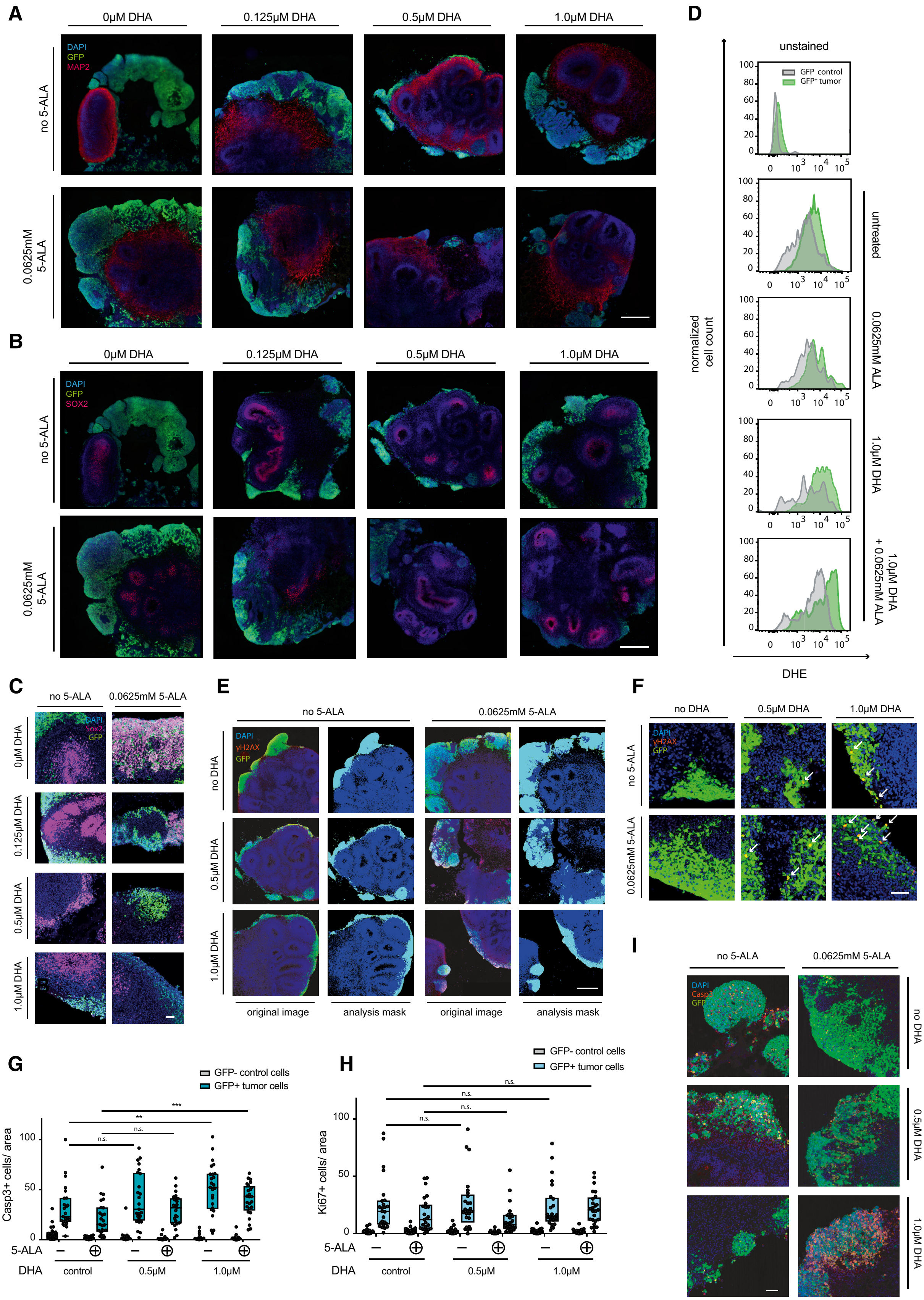

Figure EV3.

© 2023 The Authors

Figure EV4. 5-ALA and dihydroartemisinin treatment of human glioblastoma-like neoplastic organoids and patient-derived glioma cells and spheroids.

A Fluorescence images of 5-ALA- and DHA-treated cerebral tumor organoids (glioblastoma-like neoplastic organoid model). Organoids were monitored on d1, d5, d8, and d11. Scale bar 500 μm.

B Quantification of GFP-positive tumor areas of the treated brain tumor organoids. Organoids were analyzed on d8 and normalized to d1. Data are shown as box plots (25th–75th percentiles, median). The experiment was performed in quadruplicate;

C, D Patient-derived (C) high-grade glioma (VBT92) and (D) atypical teratoid rhabdoid tumor cells (VBT281) were treated with the indicated doses of cisplatin (left), VP-16 or etoposide (middle), and temozolomide (TMZ, right) for 72 h and their viability was assessed using (C, D) ATP-based CellTiter-Glo or (D) MTT/EZ4U assays. Experiments were performed in triplicate. Data are shown as mean ± SD.

E Representative brightfield images of 5-ALA- and DHA-treated patient-derived high-grade glioma spheroids (VBT92), taken on day 0, day 2, and day 3 of culture and treatment. Scale bar 500 μm.

F Viability of 5-ALA- and DHA-treated spheroids (VBT92) was assessed using the CellTiter-Glo Luminescent Assay. Experiments were performed in quadruplicates. Data are shown as mean ± SD normalized to the untreated control.

G Readhesion capacity of replated spheroids (VBT92) as assessed by crystal violet staining. Experiments were performed in quadruplicates. Data are normalized to untreated control.

H Viability of 5-ALA- and DHA-treated patient-derived atypical teratoid rhabdoid tumor (ATRT) spheroids (VBT281) was assessed using the CellTiter-Glo Luminescent AssayPooled data of three independent experiments are shown, set up in quadruplicates or duplicates each. Data are shown as means ± SD.

I Representative images of 5-ALA- and DHA-treated spheroids (VBT281), taken on day 0 and day 3 of treatment are shown. Scale bar 500 μm.

J Quantification of the readhesion capacity of replated ATRT (VBT293) spheroids as assessed by crystal violet staining and absorbance measurement. Experiments were performed in quadruplicates. Data are shown as means ± SD.

Data information: (B, F, G, H, J) n.s., nonsignificant, *$P < 0.05$, **$P < 0.01$, ***$P < 0.001$; Student's *t*-test.

© 2023 The Authors

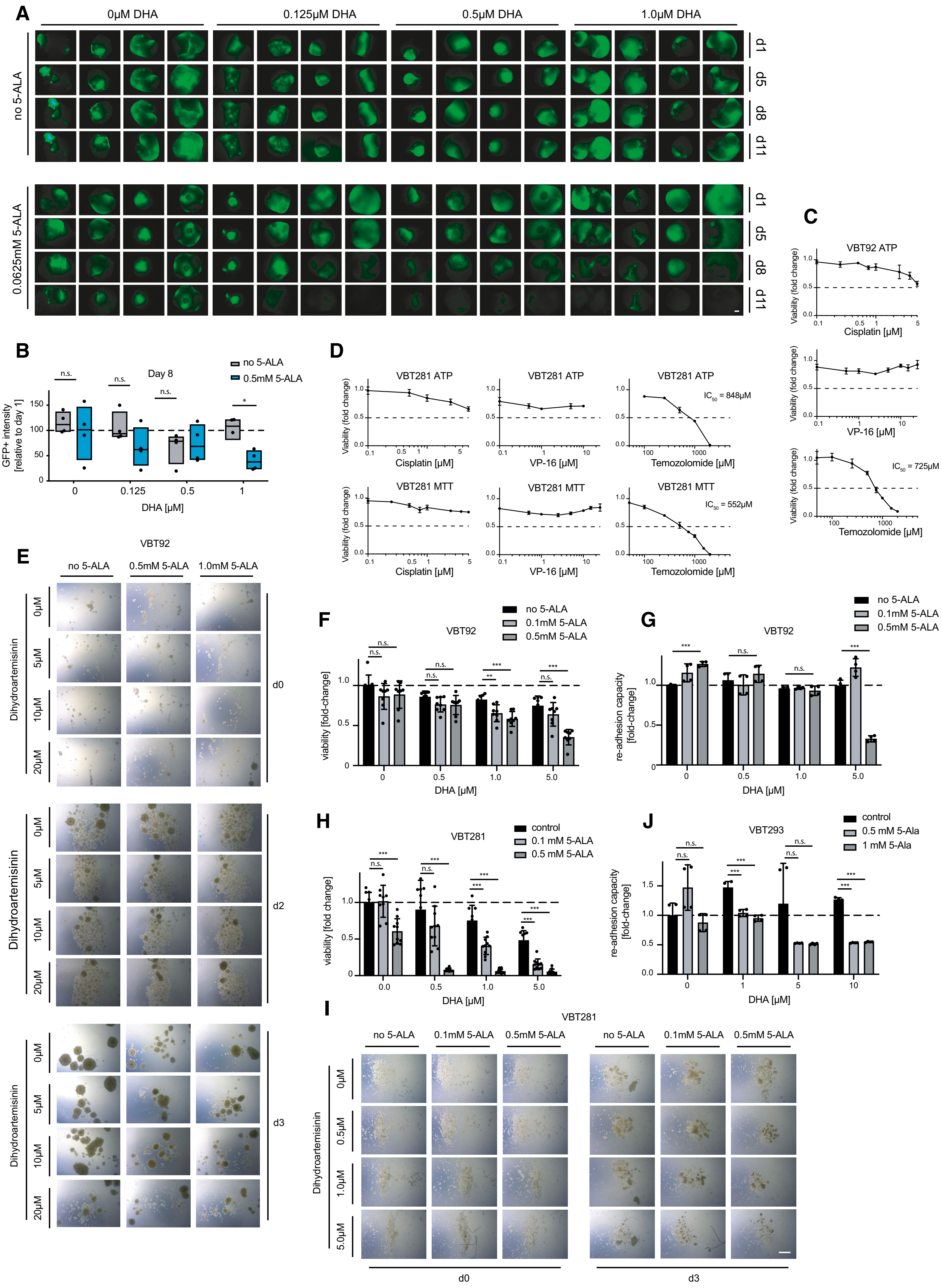

Figure EV4.

© 2023 The Authors

Figure EV5. Mechanism of Artemisinin derivatives and 5-ALA treatment of glioblastoma cells and PDX tumors in mice.

A, B Apoptosis in 5-ALA- and DHA-treated glioblastoma cells (VBT281) grown (A) in a monolayer in 2D and (B) as spheroids in 3D. Apoptosis was measured using Annexin V and PI staining and quantified by flow cytometry. The number of apoptotic cells (double positive) as a percentage of total cells is shown. Experiments were performed in triplicate and values are shown as means ± SD, one-way ANOVA followed by Bonferroni's multiple comparisons test.

C, D Lipid peroxidation in (C) VBT281 and (D) LN229 cells treated with 5-ALA and DHA. Lipid peroxidation was measured using BODIPY 581/591 C11 staining and green (from red) fluorescence shifts were determined using flow cytometry. Experiments were performed in triplicate; values are shown as means ± SD, one-way ANOVA followed by Bonferroni's multiple comparisons test.

E Representative images of mitochondrial structures in control and drug-treated glioblastoma cells (LN229) using MitoTracker Deep Red staining. Scale bar 20 μm.

F–I NAC treatment of glioblastoma cells. (F, H) VBT92 and (G, I) LN229 cells were seeded and treated with DHA and 5-ALA in presence and absence of the ROS scavenger NAC (1 mM, 1 h preincubation—clonogenic assay). Cells were stained with crystal violet and (F, G) imaged, and (H, I) absorbance was measured to determine relative viability. Experiments were performed in quadruplicate; values are shown as means ± SD.

J Body weight curves of orthotopic PDX mice treated with solvents (control), 5-ALA, artesunate (ARS), or 5-ALA plus ARS combined (same mice as in PDX main (B); $n \geq 5$). K Body weight curves of mice injected with VBT529 glioblastoma cells into the flank and brain (same mice are shown as in PDX main (C–F); $n \geq 6$).

L, M Quantification and representative pictures of the VBT531 flank tumor luminescence signal. 6–7 days after injection of 1.5×10^5 VBT531 cells into the flank, mice received the following treatments: Control (solvents) or 5-ALA plus ARS 4–5 times per week for 6 weeks via intraperitoneal injection ($n = 6$). After 2 weeks of treatment, concentrations of 5-ALA (100 mg/kg) and ARS (50 mg/kg) were increased to 120 and 60 mg/kg, respectively.

N, O Quantification and representative pictures of VBT531 brain tumor luminescence signals. 6–7 days after stereotactic intracerebral injection of 1.5×10^5 VBT531 cells, mice ($n = 6$) received treatments as described in (L and M).

P Kaplan–Meier survival curves of mice injected with VBT531 glioblastoma cells into their flanks and brains. Mice were treated with solvents (control) or 5-ALA plus ARS (same mice as in J–O).

Q Body weight curves of mice injected with VBT531 glioblastoma cells into their flanks and brains ($n = 6$).

R Quantification of porphobilinogen (PBG) and protoporphyrin IX (PPIX) levels in the unaffected brain and tumor tissue in untreated versus 5-ALA treated (120 mg/kg) glioblastoma-bearing mice ($n = 3$).

Data information: (J–R) Data are presented as means ± SEM; $*P < 0.05$, $**P < 0.01$, $***P < 0.001$; (J, K, Q) two-way ANOVA followed by Bonferroni's multiple comparisons test. (L, N, R) Student's one-tailed, unpaired t-test; (P) Log-rank (Mantel-Cox) test.

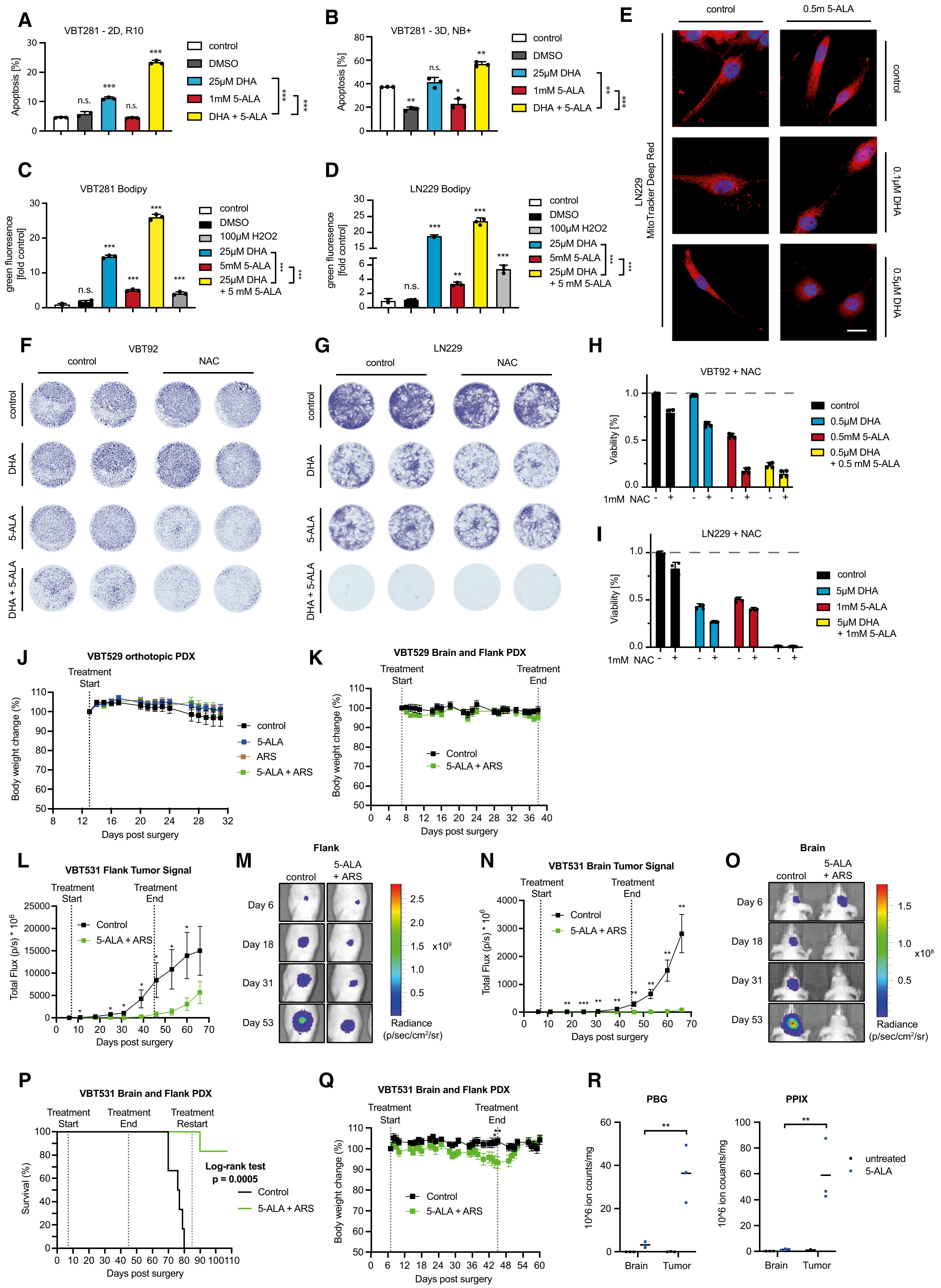

Figure EV5.

    © 2023 The Authors
